# Isoliquiritigenin Modulates Quorum Sensing-Associated Neutrophil Extracellular Trap Formation in COPD Isolate *Pseudomonas aeruginosa*

**DOI:** 10.3390/molecules31214007

**Published:** 2026-11-06

**Authors:** Honagodu Ravichandra Dhanyashree, Rakshitha Charavu, Asif Hameed, Kizhakkumkara Karthika, Adarsh Basavaraj Mynalli, Suprith Surya, Yashodhar Prabhakar Bhandary, Rajesh Padumane Shastry

**Affiliations:** 1 Division of Microbiology and Biotechnology, Yenepoya Research Centre, Yenepoya (Deemed to Be University), Deralakatte, Mangalore 575018, India; 2 Division of Cell Biology and Molecular Genetics, Yenepoya Research Centre, Yenepoya (Deemed to Be University), Deralakatte, Mangalore 575018, India; 3 Anaerobic & Translational Genomics Centre (ATGC), Yenepoya Research Centre, Yenepoya (Deemed to Be University), Deralakatte, Mangalore 575018, India; 4 *Caenorhabditis elegans* Laboratory Facility (CeLF), Yenepoya Research Centre, Yenepoya (Deemed to Be University), Deralakatte, Mangalore 575018, India; 5 Department of Respiratory Medicine, Yenepoya Medical College Hospital, Yenepoya (Deemed to Be University), Deralakatte, Mangalore 575018, India; 6 Advanced Surgical Skill ENhancement Division (ASSEND), Yenepoya (Deemed to Be University), Deralakatte, Mangalore 575018, India

**Keywords:** NETosis, isoliquiritigenin, *Pseudomonas aeruginosa*, quorum sensing, Chronic Obstructive Pulmonary Disease

## Abstract

We characterize a virulent COPD isolate, *P. aeruginosa* RS85, as a potential inducer of NET, and demonstrate the ameliorating impacts of isoliquiritigenin (ISL) on bacterial quorum sensing (QS) and neutrophil responses. Low doses of ISL exhibited anti-quorum sensing (AQS) activities on *P. aeruginosa* RS85 and PAO1, while its high dose was found to be bactericidal. Significantly high amounts of ROS and NETosis were observed in neutrophils when treated with the live cells and secretome of clinical *P. aeruginosa*, whereas *P. aeruginosa* PAO1-JP2 elicited markedly reduced responses. RS85 secretome promoted pulmonary NET formation, recruitment of inflammatory cells, alveolar destruction, and emphysema-like pathology in mouse lungs in vivo. The introduction of ISL inhibited cell- and secretome-mediated ROS and NETosis to the levels observed in PAO1-JP2. ISL treatment constrained NETosis, the co-localization of myeloperoxidase and citrullinated histone H3, lung inflammatory damage and structural damage in vivo. The molecular docking of ISL with bacterial QS (LasR and RhlR) and neutrophil NADPH oxidase assembly (p47phox and NOX2) proteins showed strong ligand binding, substantiating interference in QS signaling and ROS generation pathways. This study identifies potential QS-regulated secretory virulence in clinical *P. aeruginosa* driving NETosis, and demonstrates the ISL-mediated amelioration of NETs and bacterial QS.

## 1. Introduction

Chronic Obstructive Pulmonary Disease (COPD), a leading cause of morbidity and mortality worldwide, is a global health problem because of its chronic progression, recurrences of exacerbations, and inadequate treatments [1,2]. Although it has a strong association with cigarette smoking [3,4], COPD exacerbations are typically caused by bacterial infections that speed up disease progression, prolong hospitalization and, increase death rates, and are responsible for lung function loss [2,5,6]. Among the Gram-negative bacterial pathogens causing this disease in low- and middle-income countries such as India, *P. aeruginosa*, *Klebsiella pneumoniae*, and *Acinetobacter baumannii* are frequently isolated from COPD exacerbations [7]. Among the Gram-negative bacteria mentioned above, *P. aeruginosa* presents a substantial clinical challenge due to its intrinsic resistance to antibacterial agents, great adaptability, and persistence across airways via quorum sensing (QS)-regulated virulence factors [8]. Hence, lung injury due to *P. aeruginosa* infections is caused not only by bacterial persistence, but also by impaired host inflammatory responses.

The first-response cells of the innate immunity against invading bacteria are neutrophils, which are recruited in large quantities into the airways [9]. The infiltrated neutrophils react depending on the stimuli, most commonly with an increase in reactive oxygen species (ROS) and neutrophil extracellular traps (NETs) [10]. The formation of NETs (NETosis) is linked with reduced phagocytosis and impaired microbial clearance in COPD patients [11,12]. An increase in intracellular ROS leads to chromatin decondensation and the release of NETs [13]. Though elevated ROS is beneficial for pathogen clearance, it can also exacerbate oxidative stress and contribute to pathophysiology [13]. The excessively formed NETs are functionally dysregulated and hence promote chronic inflammation and tissue destruction [14]. In COPD, the DNA of NETs has been shown to activate cGAS/TLR9 sensing pathways, which promote the activation of NF-*κ*B, which in turn releases inflammatory mediators, such as IL-1β and IL-18 [15,16]. Recent studies have demonstrated that the suppression of such inflammatory pathways can alleviate acute lung injury [16,17]. Similarly, a recent study highlighted that the herbal preparation shuanghuanglian volatile oil-infused soluble powder (SHLO) alleviated LPS-induced pulmonary inflammation and lung injury in mice, through the modulation of TLR4/MyD88 and ERK signaling pathways [18]. In addition, the dysregulation of the miR-132/SIRT1/FoxO1 signaling axis contributes to cigarette smoke-induced pulmonary injury [19]. These findings highlight the involvement of multiple inflammatory and molecular signaling pathways in COPD progression. Thus, inhibiting excessive NETosis and associated inflammatory responses while preserving the normal defense mechanism of the host appears to be a reasonable therapeutic option.

Bacterial QS is a population density-driven intercellular communication network, which is considered as a key regulator of *P. aeruginosa* [20], coordinating the synthesis of many virulence factors involved in tissue damage, immune defense, and biofilm matrix formation [21]. The Pqs, Las, and Rhl QS systems work in conjunction to regulate the expression of proteases, toxins, pigments, and inflammatory mediators [22]. It has been found that clinical strains obtained from patients with COPD exhibit higher QS activity and virulence than experimental strains, consistent with adaptation to typical chronic airway inflammation such as COPD [23]. Recent studies have indicated that secreted bacterial virulence factors act directly on host immune cells to induce inflammation [14,21]; however, studies exploring their roles in ROS and NETosis are limited [9].

Isoliquiritigenin (ISL), a chalcone present in licorice root (*Glycyrrhiza glabra*), reportedly exhibits antioxidant, anti-inflammatory and anti-QS activities [24,25]. Earlier studies have shown that ISL can inhibit the *P. aeruginosa* QS systems (Las, Rhl and Pqs) and virulence factor production and pathogenicity in bacteria [25]. However, the possible impacts of ISL-mediated QS inhibition on host innate immunity and the neutralization of the pathogen remain unknown. As such, the infection model in mice and lung epithelial cells demonstrated that ISL increased host survival, decreased bacterial load, inhibited neutrophil infiltration, and decreased lung inflammation [26]. However, no studies to date have explored the mechanism through which QS inhibitors affect neutrophil function and ROS-mediated NETosis. In particular, the potential of ISL to modulate QS-based host–pathogen interactions and neutrophil responses under microbial infection remain unexplored. Here, ISL was hypothesized to modulate host–pathogen crosstalk by concurrently interfering with bacterial QS-regulated virulence signaling and ROS-dependent NETosis. The hypothesis was tested under simulated infection conditions in vitro (human neutrophils), followed by validation in vivo (murine model) using *P. aeruginosa* RS85, originating from the human respiratory tract as a model pathogen.

## 2. Results

### 2.1. ISL Inhibits QS-Regulated Pigment Production in Chromobacterium violaceum and P. aeruginosa

**Figure 1:**
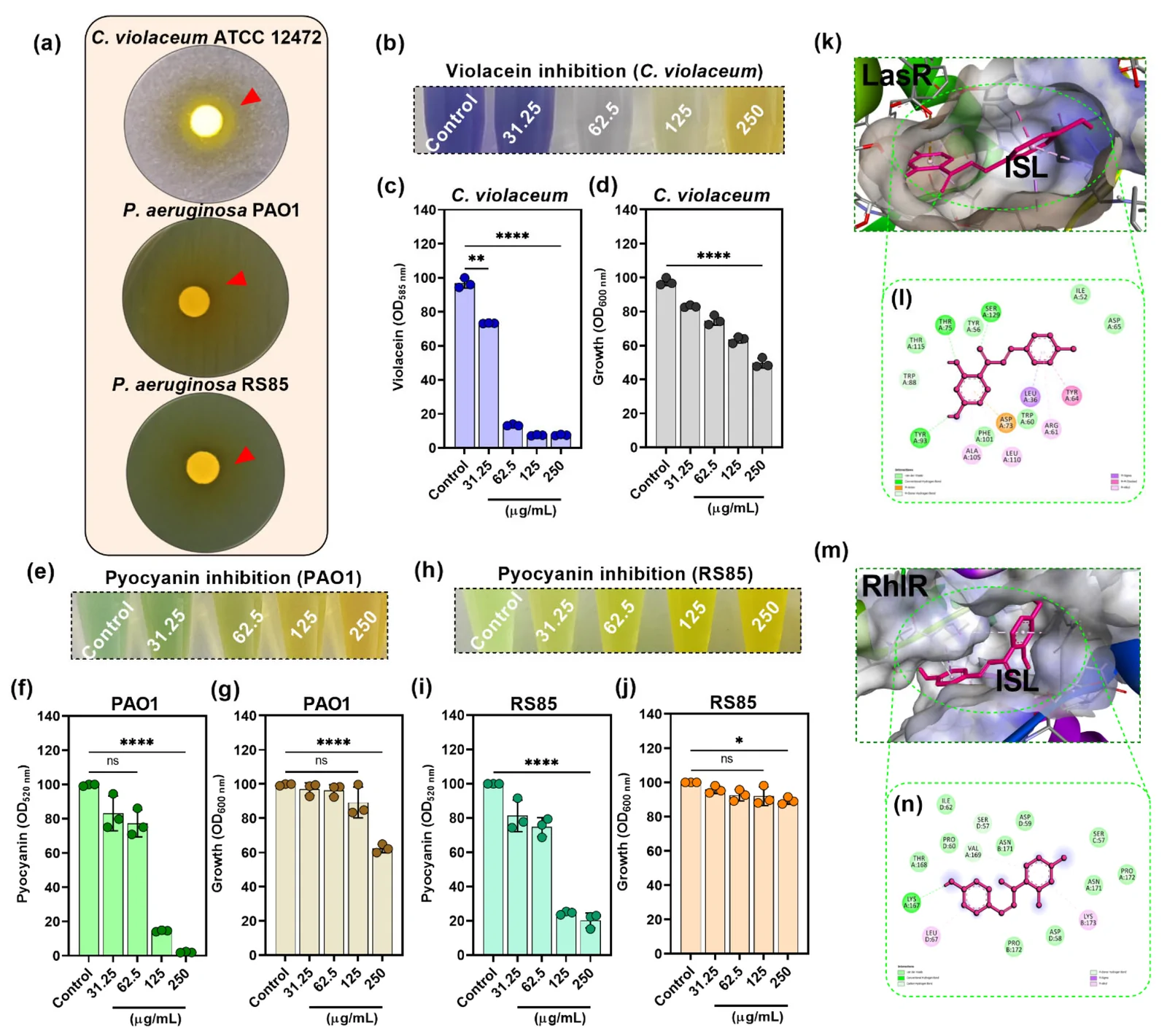
Isoliquiritigenin (ISL)-driven suppression of QS-regulated pigments in *C. violaceum* ATCC 12472, *P. aeruginosa* PAO1 and *P. aeruginosa* RS85. Anti-QS detection through the disc diffusion method (**a**). Dose-dependent inhibition of ISL on violacein and pyocyanin secretion in *C. violaceum* ATCC 12472 (**b**–**d**), *P. aeruginosa* PAO1 (**e**–**g**), and *P. aeruginosa* RS85 (**h**–**j**), respectively. Representative tube images exhibiting decreased pigment production with increasing ISL doses are shown in the inset. Data are expressed as mean ± SD (*n* = 3). Statistical significance was determined using two-way ANOVA followed by Tukey’s multiple comparisons test. Level of significance: * *p* < 0.05, ** *p* < 0.01, **** *p* < 0.0001; ns, not significant. Possible binding modes of molecular interaction of ISL with LasR and RhlR from *P. aeruginosa* quorum sensing systems. The figure illustrates the molecular docking, showing the predicted binding mode of ISL on the ligand-binding pocket of LasR (**k**) and its 2D interaction diagram (**l**). The proposed binding mode of ISL on the RhlR ligand-binding pocket (**m**) and its corresponding 2D interaction diagrams (**n**).

QS-dependent pigment production was evaluated in *C. violaceum* ATCC 12472 [27] and *P. aeruginosa* (RS85 and PAO1) to understand the possible interference of ISL with the bacterial QS system. *C. violaceum* ATCC 12472 exhibited a distinct opaque halo (8 ± 0.25) surrounding an ISL-containing disc (500 µg/disc), indicating the potential anti-quorum sensing (AQS) activity of the tested compound (Figure 1a). Similarly, a halo corresponding to pyocyanin inhibition was seen in PAO1 (16 ± 0.5) and RS85 (18 ± 0.5), suggesting consistent AQS activity by ISL. We have compared the AQS potential of ISL with 3H-furanone, a known QS inhibitor [28] using agar diffusion assays (Table 1). 3H-furanone (500 µg/disc) suppressed pigmentation in RS85 (14 ± 0.5), followed by PAO1 (12 ± 0.5) and *C. violaceum* ATCC 12472 (7 ± 0.5). Interestingly, the same dose of 3H-furanone was bactericidal against *C. violaceum* ATCC 12472, whereas this response was not observed in RS85 and PAO1, suggesting the intrinsic resistance of these strains to 3H-furanone and ISL. While solvent control DMSO showed no detectable antibacterial or AQS activities, all tested strains were susceptible to ciprofloxacin (5 µg/disc), which failed to elicit AQS activity. Thus, although ISL produced a measurable inhibition zone against *C. violaceum* ATCC 12472, it did not exhibit detectable antibacterial activity against *P. aeruginosa* PAO1 and RS85. Therefore, the reduction in pigment occurred without detectable growth inhibition, supporting the QS-modulatory effect of ISL.

We further assessed the impacts of ISL on optical cell density and QS-dependent bacterial pigment production in liquid cultures. ISL was found to inhibit violacein production and cell growth in *C. violaceum* ATCC 12472 in a dose-dependent manner (Figure 1b–d). Compared to the untreated control, cells exhibited 30% and 80% suppression in terms of violacein production when treated with 31.2 μg/mL and 62.5 μg/mL ISL, respectively, and maximum suppression (90%) occurred at 125 μg/mL (*p* < 0.0001). Optical cell density showed a significant (*p* < 0.0001) declining trend with an increasing concentration of ISL from 31.2 to 250 μg/mL. We then examined the impacts of ISL on pyocyanin production and cell growth in *P. aeruginosa* strains. A significant (*p* < 0.0001) reduction in pyocyanin production occurred in PAO1 at ≥125 μg/mL, whereas a significant (*p* < 0.0001) decline in optical density required a high dose of ISL (250 μg/mL) (Figure 1e–g). In COPD isolate RS85, the ISL-mediated suppression of pyocyanin production was dose-dependent, where significant (*p* < 0.0001) suppression was recorded at the lowest dose (31.2 μg/mL), while noticeable suppression in optical cell density occurred exclusively at the high dose (250 μg/mL, *p* < 0.05). These data imply the differential sensitivity of bacterial strains and their QS system to ISL (Figure 1h–j). These results also emphasize the effectiveness of low doses of ISL against the QS-dependent pigmentation in both *C. violaceum* ATCC 12472 and *P. aeruginosa* isolates, while high doses were confirmed to be bactericidal in action.

### 2.2. In Silico Prediction of ISL Interactions with LasR and RhlR System of P. aeruginosa

To understand the molecular mechanisms underpinning AQS driven by ISL, we subjected RS85 to genome sequencing. RS85 shared orthoANI values of 98.8% and 98.9% with *P. aeruginosa* DSM 50071T and *P. aeruginosa* PAO1, suggesting high genomic relatedness of the COPD isolate to the type strain and wild-type *P. aeruginosa*, respectively. A BLAST (https://blast.ncbi.nlm.nih.gov/Blast.cgi) search at RAST revealed 100% amino acid identity between the LasR and RhlR of RS85 and those of PAO1. The molecular interactions between ISL and the LasR/RhlR QS systems were assessed through docking studies (Table 2). ISL occupied a well-defined position within the predicted binding cavity of LasR (Figure 1k,l) and RhlR (Figure 1m,n), although the affinity of binding differed considerably. The highest docking score was obtained when ISL was docked with LasR, which gave a predicted binding energy of −11.1 kcal mol (Table 2). The docked conformation has placed the ISL molecule into the depth of the ligand-binding cavity, with the aromatic scaffold accommodated in a dense environment of interactions with peripheral residues. The hydroxyl substituents were directed towards the polar residues, and the hydrophobic backbone occupied a hydrophobic region within the cavity, producing good ligand-receptor compatibility (Figure 1k,l). A slightly different interaction profile was recorded for RhlR, where although ISL was localized at the functional region of the receptor, the predicted binding affinity was relatively lower (−7.4 kcal mol^−1^) (Figure 1m,n, Table 2). The ligand remained positioned within the binding pocket but established fewer stabilizing contacts than in the LasR complex, consistent with the reduced docking score. These observations suggest that the RhlR binding site provides a less favorable environment for ISL than that of LasR. The docking data indicate a potential interaction between ISL and the transcriptional regulators of QS signaling in general, and LasR in particular, substantiating the experimentally observed attenuation of QS-associated phenotypes post-ISL treatment.

### 2.3. LC-MS/MS for AHLs and Identification of Potential AHLs of P. aeruginosa Driving NETosis

The secretome of RS85 and PAO1 was subjected to LC-MS/MS to identify and validate corresponding N-Acyl homoserine lactones (AHLs) involved in QS signaling. N-butanoyl-L-homoserine lactone (C4-HSL, m/z: 298.64) and N-(3-oxododecanoyl)-L-homoserine lactone (3-oxo-C12-HSL, m/z: 171.19) were found to be common in RS85 and PAO1 (Table S1). Interestingly, N-(3-hydroxydecanoyl)-L-homoserine lactone (3-OH-C10-HSL, m/z: 273.38) was detected exclusively in RS85 secretome. It is important to note that the LC-MS/MS analyses were strictly qualitative, and the presence of AHLs was confirmed by their characteristic mass-to-charge (m/z) ratio and retention time with reference to authentic standards.

Neutrophils were treated with equimolar amounts of standard AHLs (100 μM) to identify the NET-inducing capacity of synthetic AHLs, irrespective of their actual concentrations in the bacterial secretome. The fluorescence microscopy of Hoechst-stained neutrophils revealed no extracellular DNA in untreated neutrophils, whereas treatment with phorbol 12-myristate 13-acetate (PMA, 50 nM), an activator of neutrophils [29], readily showed extracellular signatory DNA, reflecting the induction of NETosis (Figure S1a). All tested AHLs triggered DNA discharge in neutrophils, as made evident through the accumulation of numerous diffuse Hoechst-positive structures extracellular to the neutrophils [30]. In particular, 3-Oxo-C12 HSL treatment resulted in significantly higher (*p* < 0.0001) amounts of NETs compared with C4-HSL, C12-HSL, and PMA treatments (Figure S1a,b). It is noteworthy that C4-HSL and C12-HSL also exhibited significant amounts of NETs when compared to PMA (Figure S1a,b). Thus, 3-Oxo-C12 HSL exhibited the strongest NET-inducing capacity among the AHLs tested and identified as a potential contributor to AHL-mediated NET induction in *P. aeruginosa*. Together with the secretome experiment, these findings support the potential contribution of individual AHLs to neutrophil induction.

### 2.4. Live Bacterial Cells and Secretome of P. aeruginosa Induce NETosis and Intracellular ROS Generation in Human Neutrophils In Vitro

**Figure 2:**
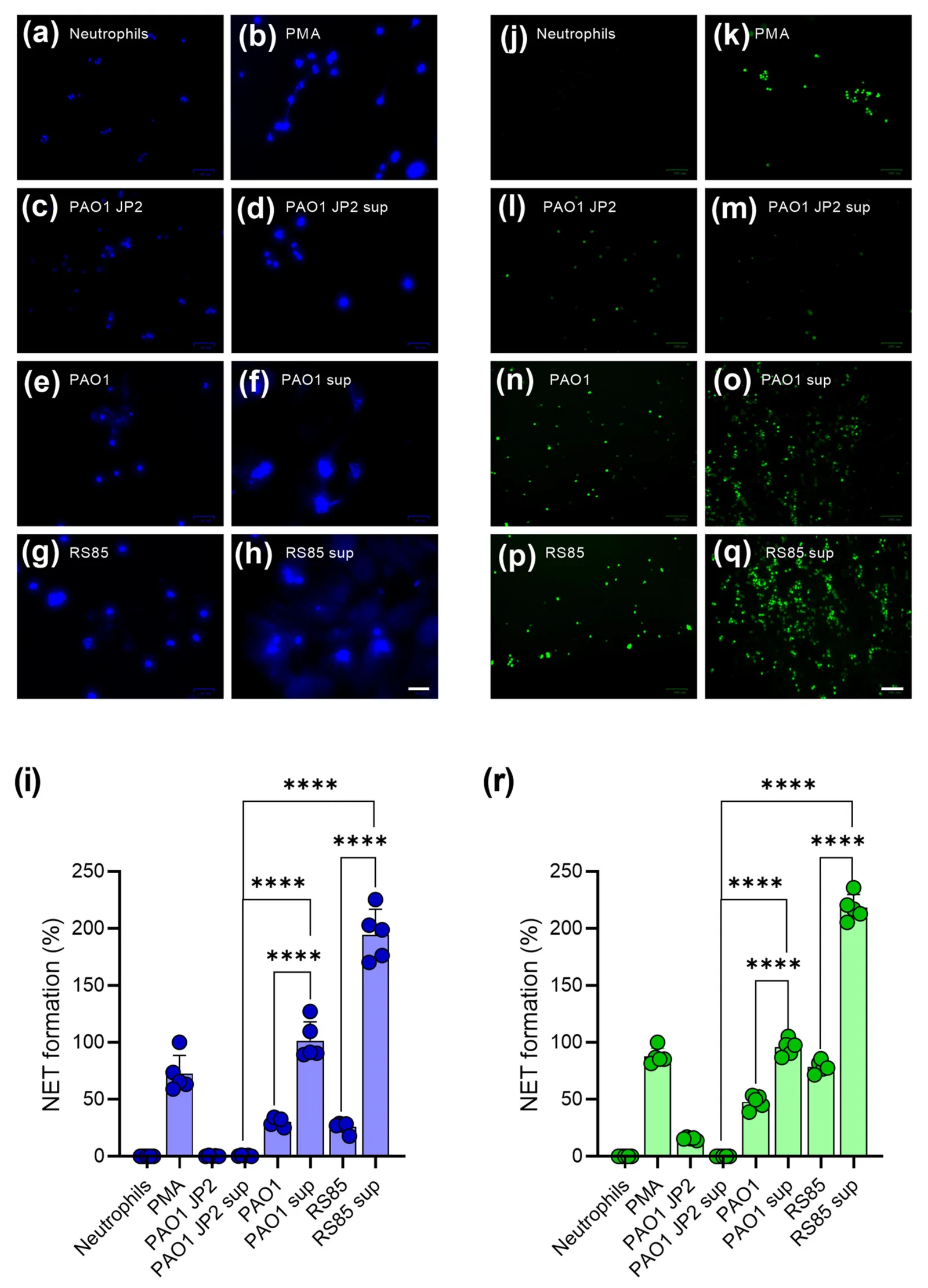
Influence of cells and secretome of *P. aeruginosa* RS85 and PAO1 on NETosis. Fluorescent microscopic view of untreated neutrophils (**a**) and neutrophils treated with PMA showing neutrophil extracellular traps (NETs) (**b**). NETs observed in neutrophils treated with cells and secretome of PAO-JP2 ((**c**,**d**), respectively), cells and secretome of PAO1 ((**e**,**f)**, respectively), and cells and secretome of RS85 (**g** and **h**, respectively); scale bar, 40 μm. The quantitative fluorescence measures of NETosis (**i**). Fluorescent microscopic view showing ROS production in bare neutrophils (**j**) and neutrophils treated with PMA (**k**), cells and secretome of PAO1-JP2 ((**l**,**m**), respectively), cells and secretome of PAO1 (**n** and **o**, respectively), and cells and secretome of RS85 (**p**,**q**), respectively); scale bar, 100 μm. The quantitative fluorescence measures for ROS (**r**) are shown. Data are presented as mean ± SD. Five regions of interest (ROIs) were analyzed per sample, with five biological replicates and two technical replicates for each experimental condition. However, ROIs and technical replicates were averaged for each donor before statistical analysis; therefore, *n* = 5 independent biological replicates (donors). Statistical significance was analyzed using one-way ANOVA followed by Tukey’s multiple comparisons test. Level of significance: **** *p* < 0.0001.

To comparatively assess the role of cells and secretory QS molecules of *P. aeruginosa* in triggering neutrophil activation [31], the freshly isolated human neutrophils were incubated either with live bacterial cells or with secretome of the wild-type strain PAO1, a LasI/RhlI-deficient mutant PAO1-JP2 and a clinical strain RS85 (Figure 2). No signatory extracellular DNA structures corresponding to NETosis were found in untreated neutrophils that were maintained under basal conditions (Figure 2a). In contrast, as expected, neutrophils treated with PMA showed the signs of NETosis, with characteristic extracellular DNA structures (Figure 2b). Neutrophils treated with PAO1-JP2 cells and secretome exhibited poor NETs (Figure 2c,d). The exposure of neutrophils to live cells of PAO1 and its secretome activated NETs, as evidenced by prominent chromatin structures around the neutrophils (Figure 2e,f). Similarly, live cells of clinical strain RS85 and its secretome induced prominent NETs (Figure 2g,h). The fluorescence intensity measurements showed no significant differences in NETs in neutrophils treated with live cells or the secretome of PAO1-JP2 (Figure 2q). In contrast, significantly higher amounts of NETs were observed in neutrophils exposed to the secretome of RS85, followed by PAO1, compared with their live cell counterparts. These findings indicate strain-specific variations in the ability of *P. aeruginosa* cells and secretome to induce NETosis, and suggest that the disruption of Las/Rhl QS systems may influence the production of bacterial factors involved in neutrophil activation [32].

Intracellular ROS production in the neutrophils is a key feature governing lytic NETosis [13]. We investigated intracellular ROS production in neutrophils challenged with live *P. aeruginosa* and its secretome using DCFH-DA as a probe. The production of ROS was negligible in untreated neutrophils (Figure 2j), while intense intracellular green fluorescence corresponding to ROS was recorded in PMA-treated neutrophils (Figure 2k). Consistent with our NETosis results, ROS production was negligible in neutrophils exposed to live cells and the secretome of PAO1-JP2 (Figure 2l,m). In contrast, live cells and secretome of PAO1 and RS85-treated neutrophils showed significant ROS production, and it is noteworthy that the ROS peaked under secretome treatment (Figure 2n–q). The fluorescence measurements, which were in line with the results of fluorescence microscopy (Figure 2r), were aligned with the earlier notion that whole bacterial cells and secretome are powerful activators of human neutrophils [33]. The reduced ROS and NET formation observed upon the exposure of neutrophils (Figure S2a–h) to PAO1-JP2 cells or secretome may be linked to the altered production of bacterial factors due to the absence of the Las/Rhl QS system. Though these findings do not strongly suggest the QS as prerequisite for ROS and NET formation, they support the association between QS-regulated secretary products and excessive NET formation.

**Figure 3:**
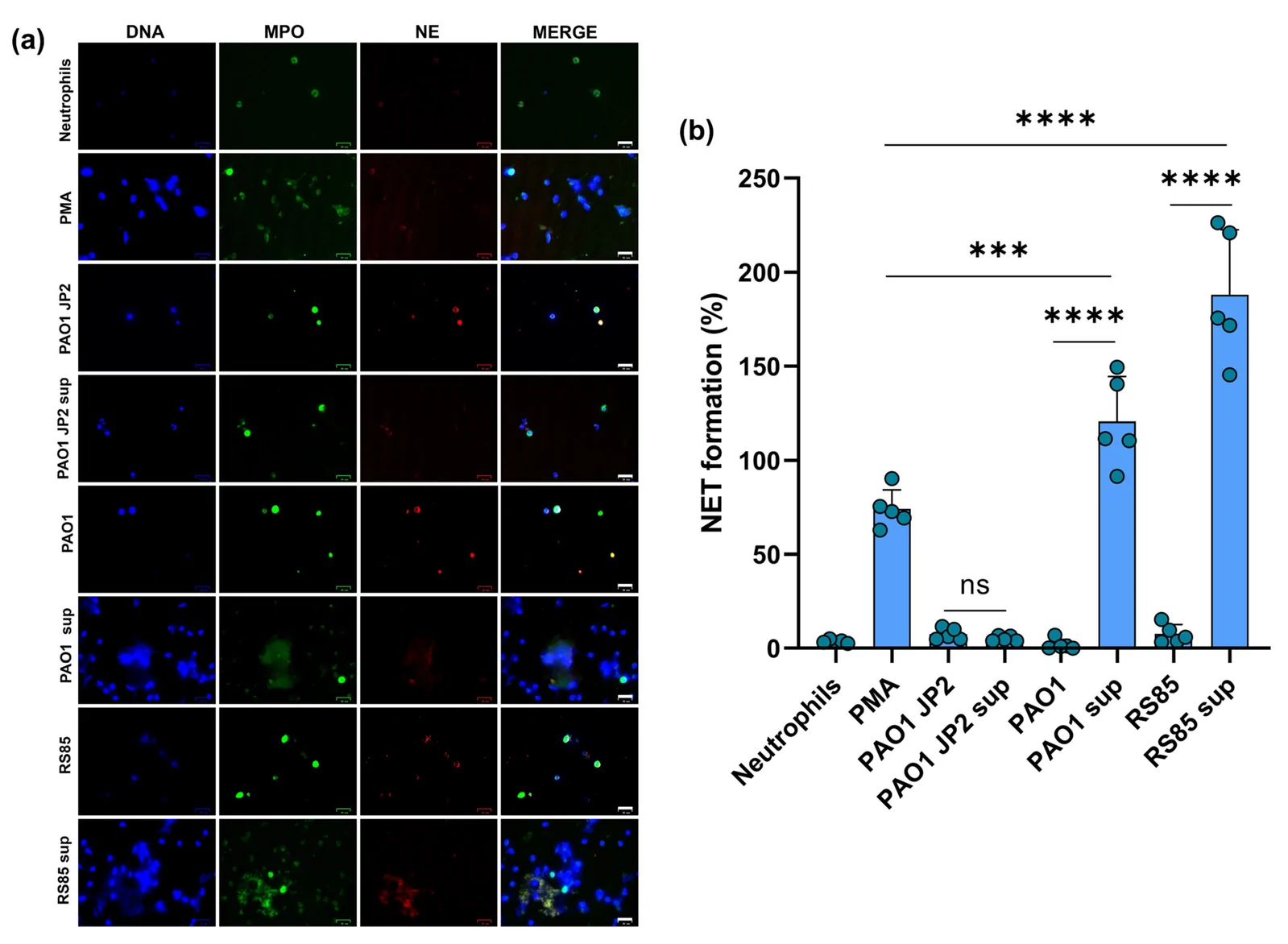
Immunofluorescence analysis of NET formation induced by bacterial stimuli. Representative fluorescence micrographs of neutrophils (**a**) stained with Hoechst (blue), myeloperoxidase (MPO; green) and neutrophil elastase (NE; red) and merged images. Untreated neutrophils served as the negative control, whereas PMA-treated neutrophils served as the positive control. Stimulation resulted in increased extracellular DNA release and the co-localization of MPO and NE with DNA, indicating NET formation. The accompanying bar graph (**b**) shows a quantitative analysis of NET-associated fluorescence intensity. Data are presented as mean ± SD. Five regions of interest (ROIs) were analyzed per sample, with five biological replicates and two technical replicates for each experimental condition. However, ROIs and technical replicates were averaged for each donor before statistical analysis; therefore, *n* = 5 independent biological replicates (donors). Statistical significance was determined using one-way ANOVA followed by Tukey’s multiple comparisons test. Level of significance: *** *p* < 0.001, **** *p* < 0.0001; ns, not significant. Scale bar, 25 µm.

We performed immunofluorescence to probe the activation of myeloperoxidase (MPO) and neutrophil elastase (NE), indicative of NET maturation [34]. Untreated neutrophils showed mild NETs with poor colocalization of MPO and NE (Figure 3a). In contrast, PMA-treated neutrophils produced extensive extracellular DNA, as evident through significant MPO and NE coating on NETs. Live cells of RS85 and PAO1 induced extracellular DNA with minimal MPO/NE colocalization. In contrast, the secretome of RS85 showed extensive decondensation of chromatin as well as a high degree of colocalization of MPO and NE in the extracellular space, followed by PAO1, whereas no such fluorescence response was observed in PAO1-JP2. Fluorescence measurements of secretome-treated neutrophils substantiated the high potency of QS molecules secreted by RS85 as compared to PAO1 in the maturation of NETs (Figure 3b). These findings suggest that strain-specific differences in bacterial secretome, influenced by the Las/Rhl QS system, contribute to NET formation along with NE and MPO localization.

Since ROS accumulation is critical during NET formation, we first assessed the intrinsic antioxidant potential of ISL. The free-radical-scavenging activity of ISL towards ABTS and DPPH was investigated using ascorbic acid as a standard in comparison with ISL (Figure S2i,j). ISL showed the potent and rapid quenching of ABTS free radicals; the percentage of inhibition achieved was greater than 75% and gradually reached about 90% at high concentrations, comparable to ascorbic acid. The IC50 value obtained for ISL to scavenge the ABTS radical was 0.897 µg/mL (Table S2), whereas for ascorbic acid it was 14.56 µg/mL. Similarly, the effect of ISL at various concentrations was also evaluated by the DPPH free radical method. ISL showed concentration-dependent activity in scavenging the DPPH radical (Figure S2j); however, compared with ascorbic acid, it exhibited a lower inhibitory effect. But at very high concentrations, ISL also produces a good (approx. 85%) inhibition of DPPH radicals. This efficient antioxidant potential of the ISL probably explains the inhibition of intracellular ROS production in *P. aeruginosa*-stimulated neutrophils, because oxidative burst is a necessary event for the activation of the ROS-dependent NETosis process. Our findings strongly support ISL’s potential to inhibit ROS and constrain NETs.

### 2.5. ISL Suppresses P. aeruginosa Cell-Induced NETosis and Oxidative Burst in Human Neutrophils

**Figure 4:**
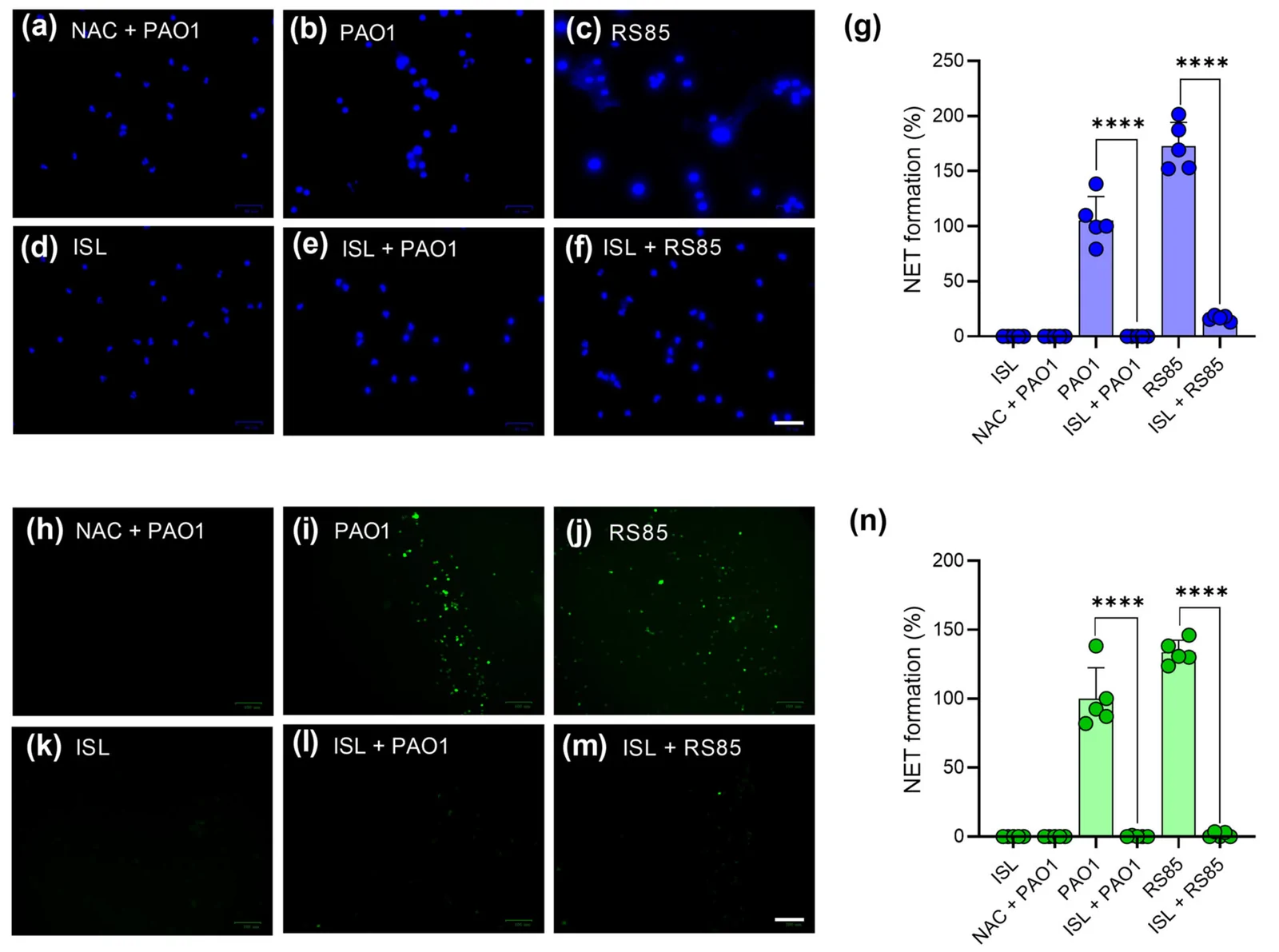
Isoliquiritigenin (ISL)-driven attenuation of *P. aeruginosa* cell-induced NETs and intracellular ROS generation in human neutrophils. Neutrophils treated with NAC and PAO1 (**a**), cells of PAO1 (**b**), cells of RS85 (**c**), ISL (**d**), a mixture of PAO1 cells and ISL (**e**) and a mixture of RS85 cells and ISL (**f**); scale bar, 40 μm. The quantitative measures of fluorescent intensity observed for NETosis (**g**). ROS production in neutrophils when treated with a mixture of PAO1 cells and NAC (**h**), cells of PAO1 (**i**), cells of RS85 (**j**), ISL, (**k**) a mixture of PAO1 cells and ISL (**l**), and a mixture of RS85 cells and ISL (**m**). Scale bar, 100 μm. The quantitative measures of fluorescent intensity observed for ROS (**n**). Data are presented as mean ± SD. Five regions of interest (ROIs) were analyzed per sample, with five biological replicates and two technical replicates for each experimental condition. However, ROIs and technical replicates were averaged for each donor before statistical analysis; therefore, *n* = 5 independent biological replicates (donors). Statistical significance was analyzed using one-way ANOVA followed by Tukey’s multiple comparisons test. Level of significance: **** *p* < 0.0001.

We investigated the possible ISL-mediated abrogation of human neutrophil activation triggered by live cells of *P. aeruginosa* [35]. Neutrophils were treated with RS85 and PAO1 in the presence and absence of ISL and screened for NETs. We first probed neutrophils in the mixed occurrence of antioxidant N-acetylcysteine (NAC, 5 mM) [36] and live cells of PAO1 for morphology and NET formation. NAC attenuated PAO1-mediated NET production, confirming that increased ROS is necessary for NETs formation (Figure 4a). As expected, neutrophils challenged with PAO1 and RS85 showed significant NETs (Figure 4b and Figure 4c, respectively). Neutrophils exclusively exposed to ISL remained intact without showcasing the NETs (Figure 4d). In contrast, the treatment of neutrophils with PAO1 and RS85 cells in the presence of ISL led to the arising of no signs of NETs (Figure 4e,f). Quantitative fluorescence data were in line with visual findings of the ISL-driven suppression of NETosis (Figure 4g).

We further probed intracellular ROS production to understand whether ISL affects oxidative burst. ROS generation being a key factor for NET formation, NAC treatment repressed neutrophils in eliciting PAO1-mediated ROS production (Figure 4h). Neutrophils treated with the PAO1 and RS85 cells showed significant ROS production (Figure 4i and Figure 4j, respectively). Neither ISL alone, nor live cells of PAO1 and RS85 with ISL input, showed significant levels of ROS (Figure 4k–m). High-intensity fluorescent signals resulting from the strong accumulation of intracellular ROS were observed when neutrophils were treated with live cells of either RS85 or PAO1 (Figure 4n). The ROS production trend was in excellent agreement with the trend observed for NETosis, where live cells of RS85 showed the maximum response, followed by PAO1 (Figure S3).

### 2.6. ISL Attenuates P. aeruginosa Secretome-Induced NETosis and Intracellular ROS Generation in Human Neutrophils

**Figure 5:**
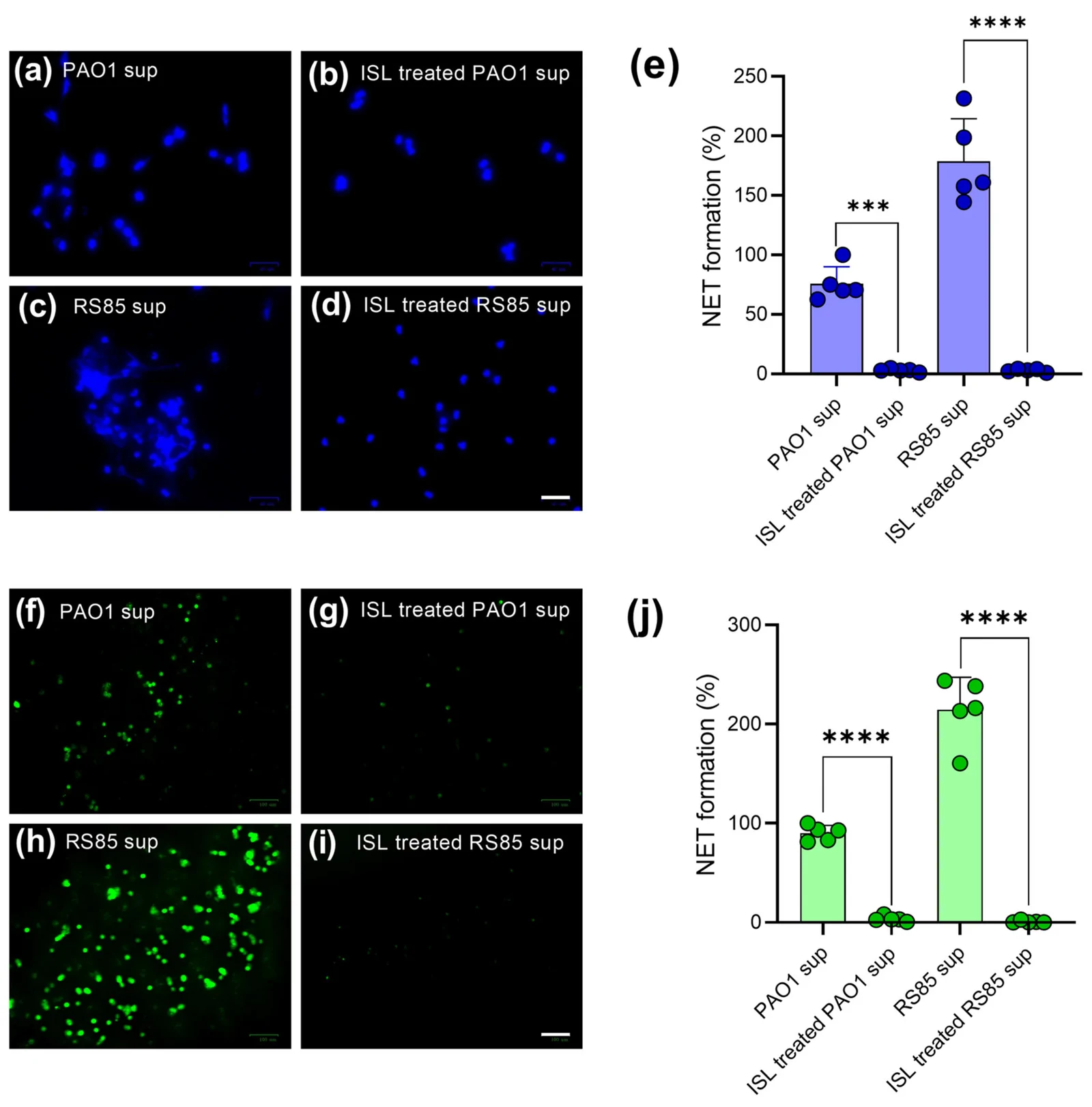
Isoliquiritigenin (ISL)-driven attenuation of *P. aeruginosa* secretome-induced NETs and intracellular ROS generation in human neutrophils. NETs when we treated neutrophils exclusively with PAO1 (**a**), PAO1 secretome and ISL mixture (**b**), RS85 secretome (**c**) and RS85 secretome and ISL mixture (**d**); scale bar, 40 μm. The quantitative fluorescence measures observed for NETosis (**e**). ROS production in neutrophils when treated exclusively with PAO1 secretome (**f**), PAO1 secretome and ISL mixture (**g**), RS85 secretome (**h**) and RS85 secretome and ISL mixture (**i**); scale bar, 100 μm. The quantitative fluorescence measures observed for ROS (**j**). Data are presented as mean ± SD. Five regions of interest (ROIs) were analyzed per sample, with five biological replicates and two technical replicates for each experimental condition. However, ROIs and technical replicates were averaged for each donor before statistical analysis; therefore, *n* = 5 independent biological replicates (donors). Statistical significance was analyzed using one-way ANOVA followed by Tukey’s multiple comparisons test. Level of significance: *** *p* < 0.001, **** *p* < 0.0001.

Human neutrophils were stimulated via the secretome of *P. aeruginosa* PAO1 and RS85, with or without ISL input, and probed for neutrophil activation. As expected, incubations with PAO1 secretome exclusively resulted in exuberant NET formation with prominent extracellular DNA fibers coating activated neutrophils (Figure 5a). NETosis was found to decline significantly in neutrophils treated with both PAO1 secretome and ISL in parallel (Figure 5b). NETosis was prominent in neutrophils treated with RS85 secretome, while ISL supplementation attenuated NET formation (Figure 5c,d). The qualitative measurement of relative fluorescent intensity associated with extracellular DNA (Figure 5e) was consistent with the microscopic results, demonstrating potential NETosis-inducing attributes of PAO1 and RS85 secretome. Thus, ISL significantly (*p* < 0.0001) diminished NET formation, indicating the effective blockade of the immune response induced by QS.

Since intracellular ROS plays a crucial role in the initiation of lytic NETosis, we determined the kinetics of intracellular ROS generation triggered by *P. aeruginosa* and implications of ISL addition using DCFH-DA as a probe. Human neutrophils treated with PAO1 secretome became highly fluorescent, reflecting oxidative burst (Figure 5f). However, upon combinatory use of ISL, PAO1 secretome treatment significantly reduced intracellular ROS fluorescence (Figure 5g). In line with this, robust intracellular ROS in neutrophils could be caught following exposure exclusively to RS85 secretome (Figure 5h), while ISL incorporation significantly reduced ROS production (Figure 5i). This trend was confirmed by fluorescent intensity measurements across the samples, where ISL significantly reduced (*p* < 0.0001) ROS generation (Figure S4). Thus, we demonstrate the potential of ISL in suppressing NETosis and intracellular ROS production as a function of PAO1 or RS85 secretome treatment. These results highlight the ability of ISL to block pro-inflammatory extracellular activities and the ROS-dependent activation of human neutrophils through AQS attributes.

### 2.7. ISL Decreased the Colocalization of MPO and NE with Neutrophil Extracellular DNA

The reduction in colocalization of MPO and NE with extracellular DNA was confirmed with immunofluorescence staining. Untreated neutrophils stimulated with *P. aeruginosa* RS85 suspension demonstrated an increased MPO and NE localization on extracellular DNA compared to *P. aeruginosa* PAO1 suspension (Figure S5). The bacterial cells, as well as the secretome present in the suspension, may contribute to the resulting NET formation, whereas ISL-pretreated neutrophils showed a reduced MPO, NE colocalization along with NET formation upon stimulation with *P. aeruginosa* PAO1, and RS85 suspension. A similar outcome was observed in NAC-pretreated neutrophils upon stimulation with *P. aeruginosa* PAO1 suspension. This indicates that ISL targets reductions in the oxidative stress responsible for NET formation. On the other hand, ISL-treated *P. aeruginosa* PAO1 and RS85 secretome significantly reduced NET formation when compared to the NETosis observed in untreated *P. aeruginosa* PAO1 and RS85 secretome.

### 2.8. ISL Did Not Reduce Neutrophil Viability

Neutrophil viability was measured by MTT and AO/EB staining assays. Neutrophils exposed to 31.25, 62.5, 125 and 250 µg/mL ISL did not show a significant concentration-dependent decrease in cell viability in the MTT assay (Figure S6), whereas in AO/EB staining, 250 µg/mL ISL showed minimal red fluorescence, indicating dead cells compared to the control. However, ISL at the selected concentration of 125 µg/mL did not significantly reduce cell viability.

### 2.9. ISL Protects P. aeruginosa Against Secretome-Induced Pulmonary NET Formation and Lung Injury In Vivo

**Figure 6:**
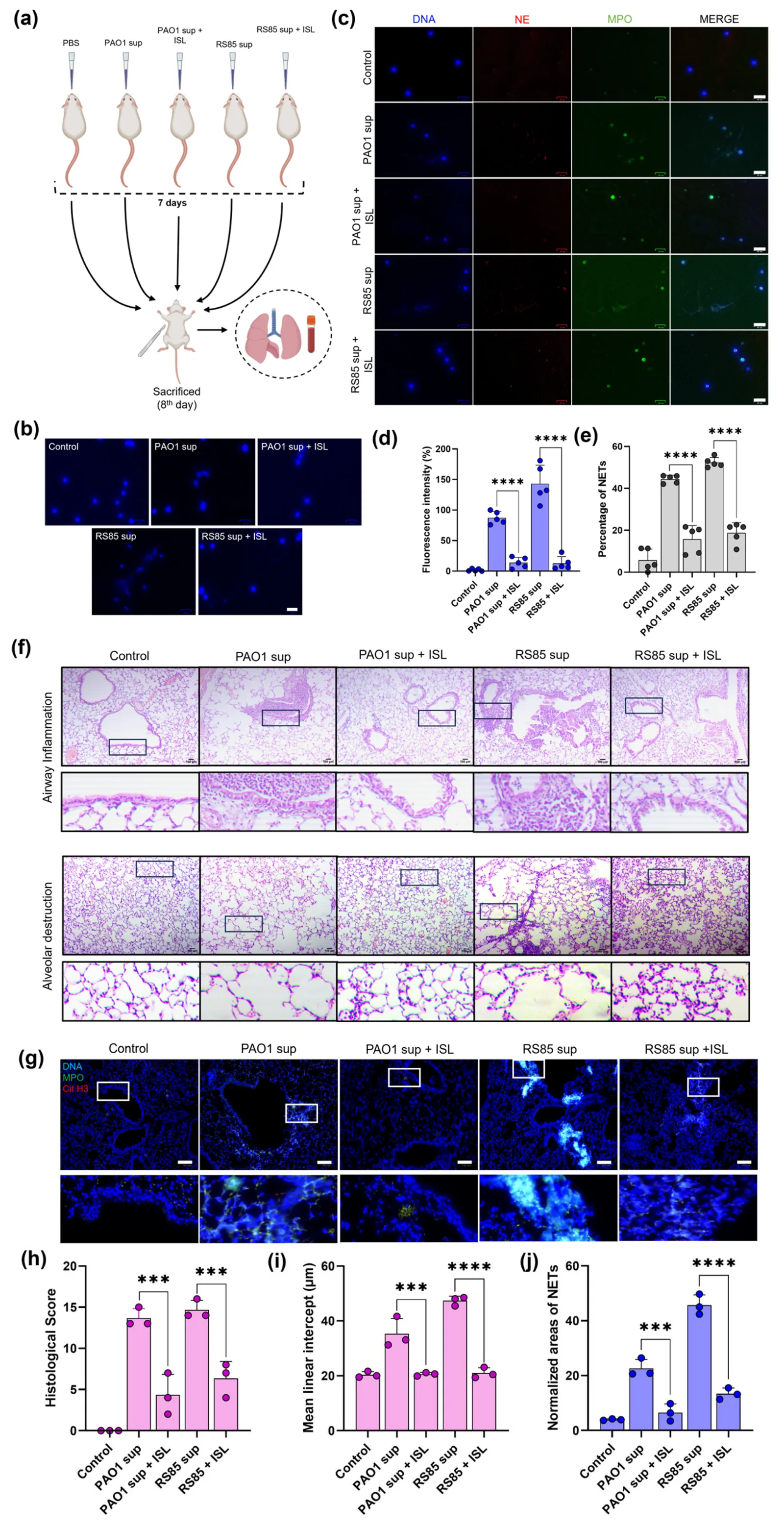
Amelioration of *Pseudomonas aeruginosa* secretome-induced NET formation, pulmonary inflammation and lung injury by isoliquiritigenin (ISL). Schematic representation of intranasal administration of PAO1 or RS85 secretome (Sup) in a murine model in the absence or presence of ISL treatment for 7 days, followed by lung and blood collection on day 8 (**a**). Fluorescence microscopy images of extracellular DNA (**b**) and immunofluorescence staining for DNA, neutrophil elastase (NE) and myeloperoxidase (MPO) (**c**); scale bar, 25 μm. Quantification of extracellular DNA fluorescence intensity (**d**) and the percentage of NET-positive cells (**e**). Hematoxylin and eosin (H&E)-stained lung sections showing airway inflammation and alveolar destruction (**f**). Immunofluorescence images of citrullinated histone H3 (Cit H3) and MPO in lung sections (**g**); scale bar, 100 μm. Histological scoring of inflammation (**h**), mean linear intercept (MLI) (**i**) and normalized area of NETs (**j**) in lung sections. Data are presented as mean ± SD. Statistical significance was analyzed using one-way ANOVA followed by Tukey’s multiple comparisons test. Level of significance: *** *p* < 0.001, **** *p* < 0.0001.

To confirm whether the AQS activity of ISL complements the innate immunity, we investigated the murine model for its ability to protect against secretome-induced pulmonary inflammation through the intranasal instillation of PAO1 and RS85 secretome for 7 days, with or without simultaneous ISL treatment (Figure 6a). Cells isolated from control mice exhibited intact condensed nuclei with minimal extracellular DNA staining, indicating negligible basal NET (Figure 6b). In contrast, exposure to RS85 and PAO1 secretome led to pronounced extracellular DNA release, as evidenced by diffused and decondensed Hoechst staining extending beyond the nuclear boundaries. These morphological changes are salient features of NETosis. RS85 secretome induced more pronounced extracellular DNA release than PAO1 secretome, whereas cells from the ISL-treated groups showed features of uninduced neutrophils, with comparatively less diffuse staining and intact nuclear morphology.

NETosis in vivo was further confirmed by the immunofluorescence staining of MPO and NE (Figure 6c,d). Low MPO and NE fluorescence was observed in neutrophils isolated from control mice. In contrast, increments in MPO and NE fluorescence, along with their co-localization with extracellular DNA, were observed in mice exposed to secretome. In the corresponding ISL-treated groups, a reduction in MPO and NE fluorescence intensity and decreased co-localization with DNA [37] were observed compared to secretome groups, indicating the effective inhibition of NETosis by ISL in vivo.

### 2.10. ISL Treatment Ameliorated Pulmonary Pathophysiology in Murine Model Exposed to P. aeruginosa Secretome

Hematoxylin and eosin (H&E) staining and histological scoring of lung sections showed that RS85 and PAO1 exposure induced marked inflammatory changes, especially increased airway inflammatory cell infiltration, the thickening and disruption of alveolar septa, and alveolar wall destruction leading to airspace enlargement [38] (Figure 6f). These structural alterations are consistent with features of lung injury commonly associated with COPD during bacterial infection [39]. The histopathological score was negligible in the control group, increased significantly to ~14 following exposure to the secretome of RS85 and PAO1, and decreased to ~5 following treatment with ISL. Morphometric analysis showed a significant increase in mean linear intercept (MLI), a marker of alveolar enlargement and emphysematous damage, following exposure to the RS85 and PAO1 secretome (Figure 6h). MLI increased from ~20 μm in controls to ~48 μm and ~35 μm in the RS85 and PAO1 groups, respectively. ISL treatment significantly reduced MLI to near baseline levels of ~21 μm, indicating that ISL is effective in reducing pulmonary inflammation and lung structural damage.

### 2.11. Reduction in NETs in the Lungs of ISL-Treated Mice Exposed to P. aeruginosa Secretome

Extensive NETosis was observed in lung sections after exposure to RS85 and PAO1 secretome, as indicated by the strong co-localization of MPO, Cit H3 and DNA NETosis in lung sections. Minimal Cit H3 and MPO signals with negligible co-localization were observed in control samples (Figure 6f,i,j). By contrast, RS85- and PAO1-secretome-treated mice showed a significant increase in Cit H3/MPO-positive extracellular structures along with DNA release, confirming enhanced NETosis. Quantitative analysis demonstrated a significant increase in NET-positive areas in RS85 (~45%) followed by PAO1 (~22%) secretome treatments. However, ISL treatment reduced NETosis to ~14% and ~7% in RS85- and PAO1-exposed lungs, respectively. These results indicate that ISL inhibits *P. aeruginosa*-induced NETosis in lung tissues.

### 2.12. ISL Exhibits Favorable Binding Interactions with p47phox and NOX2

**Figure 7:**
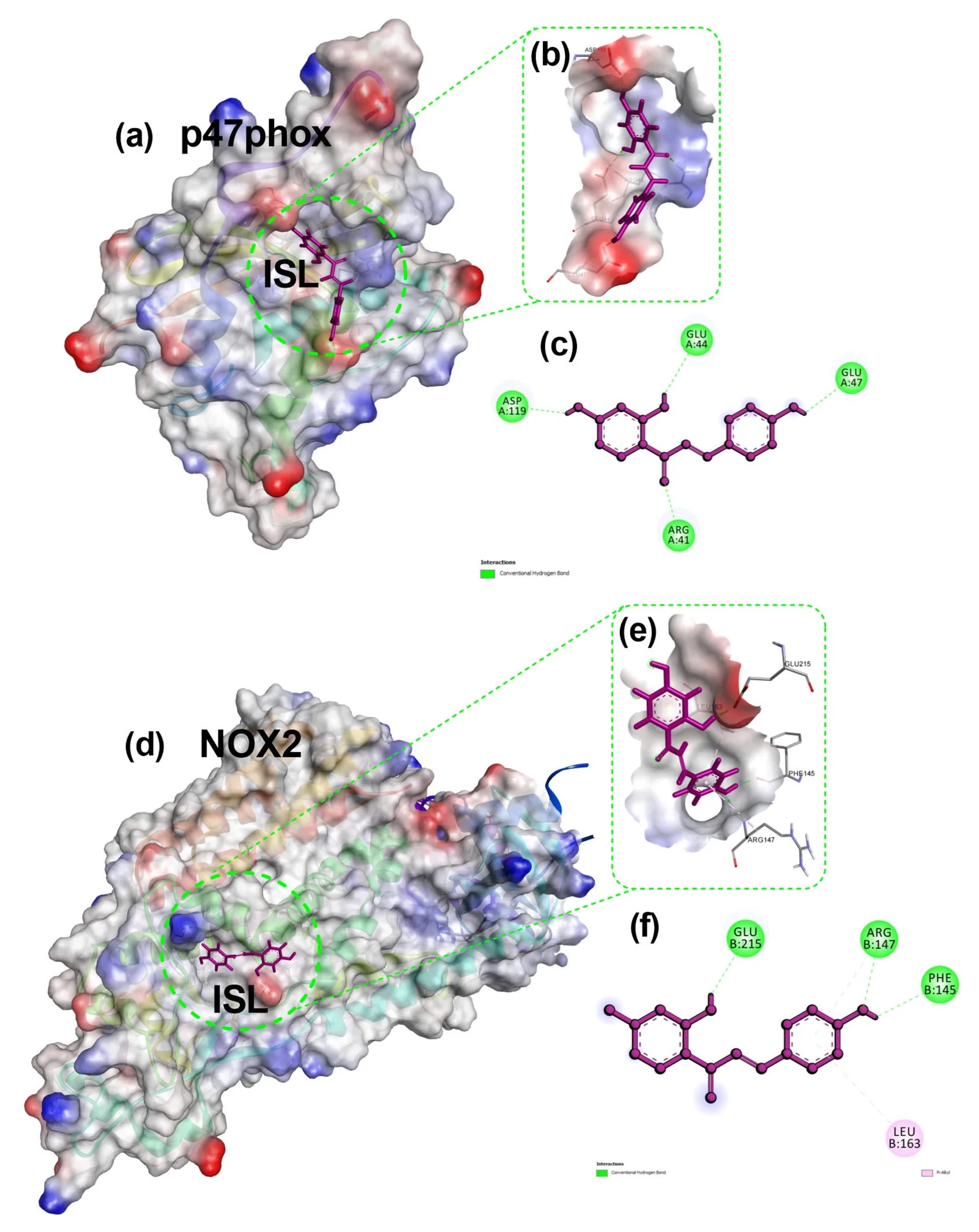
Putative molecular interactions of isoliquiritigenin (ISL) with phagocyte NADPH oxidase/NOX2 organizer (p47phox) and NADPH oxidase 2 (NOX2). Molecular docking results showing the putative binding site of ISL in p47phox (**a**,**b**) and 2D interactions (**c**). Molecular docking illustration showing the binding site of ISL in NOX2 (**d**,**e**) and 2D interactions (**f**).

To understand the molecular mechanism behind the inhibitory effect of ISL on the production of ROS and NETs, we performed molecular docking analyses of ISL against two components of phagocytic NADPH oxidase complex: the cytosolic organizing protein p47phox and the catalytic membrane subunit NOX2 [40] (Figure 7). Docking simulations predicted the binding of ISL to a specific ligand-binding pocket in both targets, indicating a stable ligand–protein interaction (Table 2). In the case of p47phox (Figure 7a–c), the docked ISL bound to a predicted binding pocket and made several conventional hydrogen bonds with ARG41, GLU44, GLU47, and ASP119, resulting in a predicted binding affinity of −7.787 kcal/mol; additionally, some hydrophobic interactions contributed to stabilizing the bound ISL. Thus, this orientation is potentially capable of hindering the ISL-mediated structural activation of p47phox and interfering with its membrane translocation, thereby impairing assembly of the active NADPH oxidase complex. Similarly, we docked ISL and NOX2 (Figure 7d–f), in which ISL was well located at the binding cavity, which was the deepest binding pocket of the catalytic subunit NOX2. Conventional hydrogen bonding with NOX2 was shown by GLU215, ARG147 and PHE145 and hydrophobic interactions (alkyl) were shown by Leu163 in the ligand–protein interaction profile of ISL and NOX2, and a predicted binding affinity of 8.062 kcal/mol was obtained. The docking data demonstrate that ISL can bind strongly to both the regulatory and catalytic domains of the NOX2 oxidase. These results provide an explanation for the experimentally observed decrease in intracellular ROS and NET formation post-ISL treatment. The interaction with p47phox and NOX2 suggests the hypothesis that ISL can inhibit oxidative burst by interfering with the assembly or enzymatic activity of NADPH oxidase, leading to the inhibition of ROS-dependent NETosis triggered by *P. aeruginosa* virulence factors. However, direct molecular targets and the underlying mechanism require experimental validation.

## 3. Discussion

**Figure 8:**
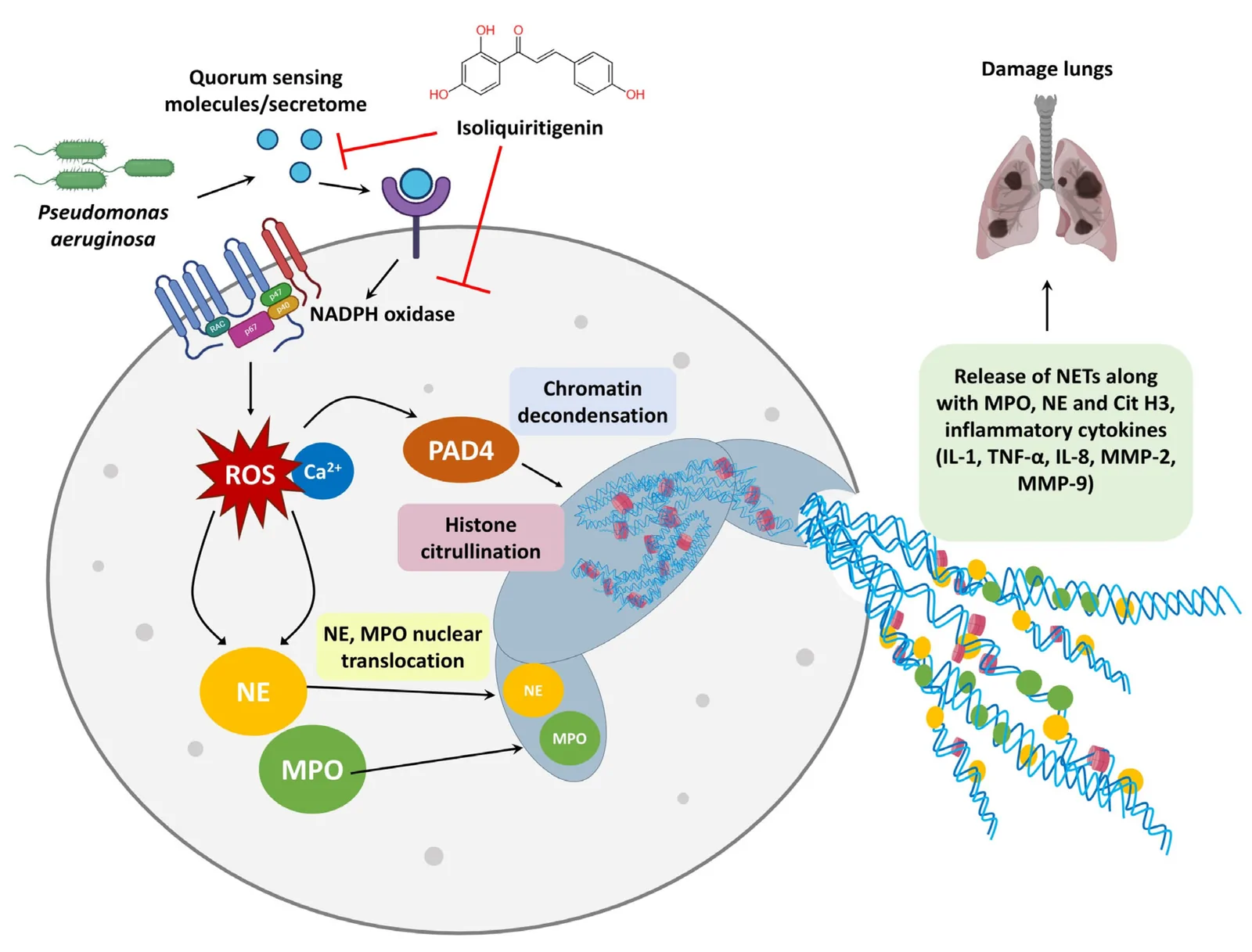
Proposed mechanism of isoliquiritigenin’s potential in *P. aeruginosa* infection and lung damage control. Schematic representation of the proposed effects of ISL in attenuating *P. aeruginosa*-induced inflammation in the lung by inhibiting quorum sensing, suppressing ROS-dependent neutrophil activation and NET production, and mitigating the inflammatory responses.

COPD, characterized by bacterial infection, often exhibits dysregulated neutrophil responses, including excessive ROS and NETosis [15]. Although the NETs are involved in microbial clearance from the airways, they are also reported to contribute to inflammation and tissue damage in COPD [10]. Currently, there is growing interest in therapeutic strategies that promote microbial clearance in COPD while limiting host damage [41,42,43]. This study demonstrates that ISL influences both bacterial virulence and neutrophil-mediated immune responses. ISL was earlier shown to play a dual role, targeting QS pathways in *P. aeruginosa* while simultaneously regulating neutrophil activation by reducing ROS production [43,44]. Though neutrophils can rely on phagocytosis for microbial killing, they tend to produce NETs [45,46]. Interestingly, NETosis has been reported to hinder the phagocytic killing of the bacteria [45,47]. The interruption of QS by ISL was demonstrated by the inhibition of the production of violacein and pyocyanin in *C. violaceum* ATCC 12472 and strains of *P. aeruginosa*, respectively, thus confirming the suppression of QS-regulated phenotypes. Consistent with our findings, a recent study demonstrated ISL’s anti-QS activity [25]. They suggest that ISL may interfere with AHL-mediated signaling pathways, thereby suppressing the expression of virulence factors [25]. Since ISL reduces the pathogenic potential of *P. aeruginosa*, there is a high chance of immunological clearance by limiting virulence-associated mechanisms that support host–pathogen interactions (Figure 8).

QS-regulated virulence factors are linked to increased NETosis [48]. In particular, pyocyanin is a redox-active pigment produced by *P. aeruginosa* [49], which functions as a non-enzymatic NADPH oxidase [50]. This leads to intracellular oxidative stress via redox cycling [48]. This enhances ROS formation and activates oxidase-dependent pathways in neutrophils, thereby promoting NETosis [50]. In addition, NETs contribute to biofilm formation by providing extracellular DNA as a structural component of the biofilm matrix, and mature biofilms can induce NETosis [51,52]. ISL has been shown to have antioxidant activity [53], directly inhibiting the oxidative burst in neutrophils during bacterial infection. This means that ISL not only inhibits pathogen-derived stimuli, but also affects innate neutrophil signaling pathways. In line with the present findings, earlier studies have demonstrated the ISL-mediated inhibition of ROS formation [54,55]. ROS generation is a critical event in NADPH oxidase-dependent NETosis; thus, decreased ROS generation reduces NETosis. ISL has been shown to decrease ROS, thus preventing neutrophils from generating NETs during *P. aeruginosa* stimulation. In agreement with the present findings, ISL has previously been shown to inhibit ROS generation [54,55]. The reduction in ROS-dependent NETosis with ISL may not be detrimental to host defense, but may rather shift the response to a more regulated and effective immune response. Thus, controlling excessive NETosis without eliminating antimicrobial activity could be a useful strategy to reduce inflammation while retaining pathogen clearance.

The in vitro findings were further supported by in vivo studies conducted in a murine model. The exposure to secretome of *P. aeruginosa* strains resulted in significant NETosis and pulmonary inflammation. ISL treatment significantly ameliorated NETosis in the lung, demonstrating its ability to modulate neutrophil responses under physiological conditions. This reduction in NETs correlated with improved histopathological features, indicating ISL’s effectiveness in alleviating tissue damage caused by hyperactive neutrophils. The anti-inflammatory effect of ISL is thought to be due to its ability to modulate key intracellular signaling pathways involved in inflammation and oxidative stress [15]. The consistency between in vitro and in vivo observations strengthen the overall conclusion that ISL plays a protective role in regulating infection-induced inflammatory responses. An important aspect of the present study is to focus on targeting bacterial virulence rather than their viability. Traditional antimicrobial therapies are mainly directed at killing pathogens, which often leads to the development of antimicrobial resistance due to high selective pressure [56]. On the other hand, QS inhibition is an alternative approach that reduces pathogenicity and reduces the risk of resistance development. Similarly, clinical studies have shown that inhaled corticosteroid therapy for COPD is associated with an increased likelihood of *P. aeruginosa* isolation from lower respiratory tract samples [23,57].

The in vitro and in vivo findings were further supported by in silico docking studies. ISL established multiple hydrogen bonds with residues in p47phox, which are critically important for defining binding specificity and binding strength. These findings indicate that ISL may interact with the p47phox-binding cavity, potentially influencing its activation and translocation to the membrane, thereby contributing to reduced NADPH oxidase activation by disrupting its conformation. The phosphorylation of p47phox causes conformational changes required for the assembly of the NADPH oxidase complex [58]. This could suppress superoxide anion production, thereby blocking the oxidative burst. ISL is also bound to the catalytic component NOX2, forming several hydrogen bonds and a hydrophobic interaction with Leu163, suggesting that ISL can bind strongly to NOX2 and potentially interfere with its catalytic activity and/or structural integrity. It is also important to note that the inhibition of NOX2 at different stages of the assembly and activation cascade has a better suppressive effect on ROS production [59]. So, the dual targeting of both the regulatory and catalytic subunits of ISL on NOX2 could be more effective in inhibiting NOX2 activity than a single-target agent. Therefore, the predicted interactions of ISL with both p47phox and NOX2 provide a possible molecular basis for the observed reduction in ROS and NET formation. However, these docking results represent the predicted protein–ligand interactions and do not establish the direct inhibition of p47phox or NOX2. The study showed a clear association between ISL treatment and reduced NETosis, but the precise molecular mechanisms underlying this effect require further investigation. In summary, this study reports the ability of the COPD-derived clinical isolate *P. aeruginosa* RS85 to induce NETosis in a QS-regulated manner, and indicates that ISL can attenuate QS-dependent virulence in *P. aeruginosa* and neutrophil-mediated inflammatory responses, including ROS generation and NET formation (Figure 8).

## 4. Methods

### 4.1. Study Approvals

This study was approved by the Yenepoya Ethics Committee-2, Yenepoya (Deemed to be University) (Protocol number YEC2/2025/133) for the collection of blood samples. The mouse model was approved by the Yenepoya University Institutional Animal Ethics Committee (Approval number: YU/IAEC/P03/2025).

### 4.2. Chemicals

Isoliquiritigenin was purchased from Tokyo Chemical Industry (TCI), India. N-acetyl cysteine (NAC) was procured from Himedia Laboratories Pvt. Ltd., Thane (West), India. 3H-furanone and phorbol 12-myristate 13-acetate (PMA) were procured from Sigma-Aldrich, Bangalore, India and Thermo Scientific, Waltham, MA, USA, respectively. AHL molecules 3-Oxo-C12 HSL, C12-HSL, and C4-HSL were from Sigma-Aldrich, Bangalore, India.

### 4.3. Bacterial Strains, Reagents and Culture Conditions

*P. aeruginosa* RS85 was isolated from the sputum sample of a COPD patient (Ethical approval No. YEC-1/2021/029) [60] and *P. aeruginosa* PAO1 was revived from our laboratory −80 °C storage. *P. aeruginosa* PAO1-JP2, a *lasI/rhlI* mutant, served as the QS-deficient control [61]. All three strains were cultured using Luria-Bertani (LB) broth. *C. violaceum* ATCC 12472, a biosensor strain for QS and violacein pigment production, was cultured in Tryptic Soy (TS) broth. Bacterial cultures were incubated for 24 h in a shaking incubator at 32 °C for *C. violaceum* and 37 °C for *P. aeruginosa*.

### 4.4. Genome Sequencing and Bioinformatics

The genome of *P. aeruginosa* RS85 was sequenced using the Illumina MiSeq platform as per earlier description [62]. The genome was uploaded to RAST [63] for gene annotation, followed by manual screening of the acyl-homoserine lactone-binding transcriptional activator proteins LasR and RhlR using the corresponding reference sequences for PAO1 retrieved from UniProt. Furthermore, to assess the molecular binding affinity of ISL to *P. aeruginosa*, docking studies were performed using the previously reported method for LasR and RhlR [64].

### 4.5. QS Inhibition Assay

QS inhibition was assessed though disc diffusion as described previously [65]. Overnight cultures of *C. violaceum* ATCC 12472 and PAO1, adjusted to 0.5 MacFarland (equivalent to 1 × 10^8^ CFU/mL), were spread-plated onto TS and LB agar, respectively. A sterile disc impregnated with ISL (500 µg/mL) was dried at 37 °C for 1 h and placed on the agar surface. 3H-furanone, a known anti-QS compound, was considered as a positive control for QS activity with the same concentration as that of ISL. Ciprofloxacin disc (5 µg, Himedia, India) was used as a positive control for antibacterial activity. TSA plates were incubated at 32 °C for optimum violacein pigment production by *C. violaceum* ATCC 12472, whereas LBA plates were incubated at 37 °C for 24 h. QS inhibition was identified by the formation of a non-pigmented, colorless zone with viable cells, clearly distinct from growth inhibition. The zones (mm) of QS and bacterial inhibition were measured.

### 4.6. Quantification of Violacein

An overnight culture of *C. violaceum* ATCC 12472 was inoculated into freshly prepared TS broth containing ISL at concentrations ranging from 31.2 to 500 µg/mL in conical flasks. The flasks were incubated at 32 °C for 24 h with continuous shaking at 120 rpm. After incubation, violacein was extracted by centrifugation at 13,000 rpm for 10 min; the pellet was then resuspended in DMSO, vortexed, and centrifuged again under identical conditions. The absorbance of the violacein-containing supernatant was measured spectrophotometrically at 585 nm [66].

### 4.7. Quantification of Pyocyanin

Quantification of pyocyanin was performed using the chloroform–HCl extraction method previously described [60]. Briefly, an overnight culture of PAO1 was inoculated into sterile LB broth supplemented with ISL at concentrations ranging from 31.2 to 500 µg/mL in conical flasks and incubated at 37 °C for 24 h with continuous shaking at 120 rpm. After incubation, cultures were centrifuged at 8000 rpm for 10 min to obtain a cell-free supernatant containing the pigment. Pyocyanin was extracted from the supernatant with an equal volume of chloroform, followed by the addition of 0.2 M hydrochloric acid, which formed a pinkish-red layer. The absorbance of this pinkish-red chromophore was recorded at 520 nm, while the pellet was resuspended in distilled water, and bacterial growth was quantified at 600 nm.

### 4.8. Identification of N-Acyl Homoserine Lactones (AHLs) by Liquid Chromatography–Mass Spectrometry

AHLs were extracted from RS85 and PAO1 using the dichloromethane (DCM) extraction method with minor modifications [67]. Briefly, *P. aeruginosa* strains were grown in LB broth at 37 °C overnight with continuous shaking. The bacterial suspension was centrifuged at 8000 rpm for 10 min. The cell-free supernatant was collected and an equal volume of DCM was added. The mixture was shaken vigorously for about 5 min, then centrifuged at 10,000 rpm for 5 min. The lower DCM layer was collected in a clean, dry glass conical flask. This process was repeated three times. DCM layers were combined and evaporated to dryness. The dried extracts were re-suspended in 200 µL HPLC-grade methanol.

The LC-MS/MS analysis of AHL extracts was performed as per a previously reported method with minor modifications [68] using an Agilent HPLC system coupled to a Waters Micromass Quattro Micro API triple quadrupole mass spectrometer (Waters Corporation, Milford, MA, USA). Samples were prepared using methanol, acetonitrile, water, 0.1% formic acid, and trifluoroacetic acid (TFA) as diluents. Chromatographic separation was performed on a Waters XBridge C18 column (50 × 4.6 mm, 3.5 µm particle size; Waters Corporation, Wexford, Ireland). The mobile phase consisted of 0.1% formic acid in water (solvent A) and acetonitrile, of MS grade (solvent B). Elution was performed using a gradient program at a flow rate of 1.2 mL/min. The gradient was as follows: 85% A/15% B at 0.01 min, linearly changed to 25% A/75% B at 6.0 min, held until 8.0 min, returned to 85% A/15% B at 11.0 min, maintained until 15.0 min. The total run time was 15.01 min. Detection was performed using a UV–visible detector. Mass spectrometric analysis was conducted using an electrospray ionization (ESI) source operating in triple quadrupole (QqQ) MS/MS mode. Instrument control and data acquisition were performed using Mass Lynx software (Version 4.2). The capillary voltage was set to 3.45 kV, cone voltage to 33 V, and extractor voltage to 3.0 V. The source temperature was maintained at 110 °C, and the desolvation temperature was set to 350 °C. Nitrogen was used as the desolvation gas at a flow rate of 750 L/h and a cone gas flow of 50 L/h. The scan time was set to 0.2 s.

### 4.9. Blood Sample Collection and Neutrophil Isolation

In total, 3 mL of peripheral blood was collected from healthy individuals in EDTA tubes. A total of 18 independent donors were included in the study, with each donor considered an independent biological replicate. Each *in vitro* experiment was performed using neutrophils isolated from five biological replicates and two technical replicates. Technical replicates were performed for each biological replicate (donor) to assess measurement variability. Neutrophils were isolated from the blood samples using EasySep™ Human Neutrophil Isolation Kit (StemCell Technology, Vancouver, BC, Canada) as per the manufacturer’s instructions. Briefly, the Isolation Cocktail was added at 50 µL/mL of sample, followed by an equal volume of RapidSpheres™. After incubation at room temperature for 5 min, the volume was adjusted to 4 mL with the recommended medium. The suspension was placed in the EasySep™ cell separation magnet for 5 min at room temperature. Collected neutrophils were quantified in a Neubauer chamber using a hemocytometer.

### 4.10. Preparation of P. aeruginosa Inoculum and Secretome

Overnight grown culture of RS85, PAO1 and PAO1-JP2 were adjusted to an optical density (OD) of 0.5 at 600 nm (1 × 10^8^ CFU/mL) and used as inoculum for pretreatment experiments. For the preparation of cell-free supernatant (secretome), overnight cultures were centrifuged at 8000 rpm for 10 min at 4 °C. The secretome was collected and filtered through a 0.22 µm syringe filter to remove residual bacterial cells. The pellet was washed twice and resuspended in phosphate-buffered saline (PBS), and the OD_600_ was adjusted to 0.5 McFarland units for testing.

### 4.11. Effect of QS-Associated Bacterial Secretome on NETosis

Neutrophils were seeded at a density of 1 × 10^5^ cells/well in 96 well plates in RPMI-1640 medium and allowed to adhere for 20 min at 37 °C [69]. Washed bacterial pellets of RS85, PAO1 and PAO1-JP2 were added to the neutrophils at a multiplicity of infection (MOI) of 1:5. To evaluate the effect of QS-associated secretory factors on NETosis, neutrophils were stimulated with 1% bacterial secretome. Cells were incubated for 3 h at 37 °C in a humidified atmosphere containing 5% CO_2_ to facilitate NETosis. Unstimulated neutrophils and neutrophils treated with 50 nM phorbol 12-myristate 13-acetate (PMA, Thermo Scientific, USA) were taken as negative and positive controls, respectively. Following incubation, cells were fixed with 4% paraformaldehyde (PFA) for 15 min and stained with 5 µM Hoechst dye (Himedia, India) for 15 min at room temperature in the dark. NETs were visualized using ZOE^TM^ fluorescent cell imager (Bio-Rad Laboratories, Inc., Hercules, CA, USA) and subsequently quantified using ImageJ software version 1.53k.

### 4.12. Treatment of Human Neutrophils with Synthetic AHLs

Freshly isolated human neutrophils were seeded in RPMI 1640 medium at a density of 1 × 10^5^ cells/well. The cells were allowed to adhere for 20 min at room temperature. Neutrophils were treated with 100 µM of the synthetic QS molecules, N-butanoyl-L-homoserine lactone (C4-HSL), N-dodecanoyl-L-homoserine lactone (C12-HSL) and N-(3-oxodedecanoyl)-L-homoserine lactone (3-oxo-C12-HSL), and incubated for 3 h at 37 °C in 5% CO_2_. Untreated neutrophils and phorbol 12-myristate 13-acetate (PMA, 50 nM)-treated neutrophils served as the negative and positive controls, respectively. Following the incubation, the cells were fixed with 4% paraformaldehyde (PFA) to preserve the structure of NETs and stained with 5 µM Hoechst 33342 stain for 15 min. Fluorescent images were acquired using ZOE^TM^ fluorescent imager. NET formation was quantified by measuring fluorescence using ImageJ software.

### 4.13. Effect of Combined Treatment of ISL and P. aeruginosa on NETosis

PAO1 and RS85 cultures were treated with ISL (125 µg/mL) overnight prior to co-incubation with neutrophils. Following ISL treatment, bacterial cultures were centrifuged at 8000 rpm for 10 min to separate the bacterial pellet from secretome. Seeded neutrophils (1 × 10^5^ cells/well) were exposed to either bacterial cells (MOI 1:5) or 1% (v/v) filter-sterilized secretome. Cells were incubated under standard conditions (37 °C, 5% CO_2_) for 3 h to induce NETosis. Controls included neutrophils exposed to untreated bacteria (MOI 1:5) or 1% culture supernatant, with 50 nM PMA as a positive NETosis; parallel conditions used PAO1-JP2 cells (MOI 1:5) or their secretome. After stimulation, cells were fixed with 4% PFA and stained with 5 µM Hoechst stain, and NETs were visualized and quantified.

### 4.14. Effect of ISL Pretreatment on NETosis

To assess the host-directed antioxidant effect of ISL, seeded neutrophils (1 × 10^5^ cells/well) were pretreated with ISL at a concentration of 125 µg/mL for 30 min before stimulation. For bacterial stimulation, RS85 and PAO1 were added at MOI 1:5 and cells were incubated for 3 h at 37 °C with 5% CO_2_ to induce NETosis. Control groups included unstimulated neutrophils, neutrophils treated with ISL alone, PMA (50 nM), and the N-acetyl cysteine (NAC, Sigma-Aldrich)-pretreated PAO1-stimulated group. After incubation, cells were fixed with 4% PFA, stained with Hoechst dye (5 µM), and assessed for NETs through fluorescence microscopy.

### 4.15. Intracellular ROS Measurement in Neutrophils

Intracellular ROS generation in neutrophils was assessed using the fluorescent probe 2′,7′-dichlorofluorescin diacetate (DCFH-DA, Himedia, India) [14]. The experimental and control groups were maintained as described for NETosis assays, including ISL pretreatment, bacterial stimulation, and appropriate controls. Briefly, seeded neutrophils (1 × 10^5^ cells/well) were subjected to respective treatments for 30 min, followed by incubation with 10 µM DCFH-DA for 30 min at 37 °C in the dark. After incubation, the cells were washed with PBS to remove excess dye. N-acetyl cysteine (NAC) was used as a ROS-inhibitory control. Fluorescence generated due to the ROS-mediated oxidation of DCFH-DA was visualized using the ZOE^TM^ fluorescent imager, and fluorescent intensity was quantified using ImageJ software.

### 4.16. Immunofluorescence Staining

To assess NET formation following bacterial stimulation and further evaluate the effects of ISL intervention, immunofluorescence staining was performed as previously reported [70]. Briefly, 1 × 10^5^ cells/well of isolated neutrophil suspended in PBS were seeded in RPMI-1640 medium and allowed to adhere to a 96-well plate. Following the adhesion, cells were stimulated by washed bacterial pellets of *P. aeruginosa* RS85, PAO1 and PAO1-JP2 at an MOI 1:5 or with 1% bacterial secretome. To evaluate the effects of ISL, neutrophils were stimulated with secretomes from ISL-treated bacterial cultures or pretreated with ISL before exposure to bacterial suspensions. Unstimulated neutrophils and neutrophils treated with 50 nM PMA served as negative and positive controls, respectively. The cells were incubated for 3 h at 37 °C in a humidified atmosphere containing 5% CO_2_. Following incubation, the cells were fixed with 4% PFA for 15 min and washed with PBS. Subsequently, cells were permeabilized with 0.3% Triton X-100 and blocked using 5% bovine serum albumin (BSA) for 1 h at room temperature to prevent non-specific binding. For NET assessment, cells were incubated with goat anti-myeloperoxidase (MPO) antibody (1:100; R&D Systems, Minneapolis, MN, USA; AF3667) and rabbit anti-NE antibody (1:100; Affinity Biosciences, Cincinnati, OH, USA; AF-0010) overnight at 4 °C. After primary antibody incubation, cells were washed and incubated with secondary antibodies, Alexa Fluor 488 donkey anti-goat and Alexa Fluor 647 donkey anti-rabbit (1:1000, Invitrogen™, Thermo Fisher Scientific, Inc., Waltham, MA, USA). DNA was counterstained using Hoechst dye (5 µM). Images were acquired using the ZOE^TM^ fluorescent cell imager. Fluorescence intensity was quantified using ImageJ software.

### 4.17. Cytotoxicity Assays

ISL cytotoxic effect was evaluated using the colorimetric 3-(4,5-dimethylthiazol-2-yl) diphenyltetrazolium bromide (MTT) reduction [71] and acridine orange/ethidium bromide (AO/EB) staining assays [72]. For the MTT assay, neutrophils were seeded at a density of 1 × 10^5^/well in a 96-well plate in RPMI-1640 medium and allowed to adhere for 20 min at 37 °C. The adhered cells were exposed to different concentrations of ISL (31.25, 62.5, 125 and 250 µg/mL) for 3 h at 37 °C + 5% CO_2_. The supernatant was removed from each well, and the cells were washed with phosphate-buffered saline (PBS). The cells were incubated with 0.5 mg/mL MTT in a fresh medium for 2 h at 37 °C + 5% CO_2_. The formed crystalline formazan was dissolved using 100 µL DMSO. The absorbance was then measured at 570 nm using a FLUOstar Omega (version 5.15) microplate reader (BMG LABTECH GmbH, Ortenberg, Germany). For AO/EB staining assay (live/dead assay), neutrophils (1 × 10^5^/well) were exposed to different concentrations of ISL (31.25, 62.5, 125 and 250 µg/mL) and incubated for 3 h at 37 °C + 5% CO_2_. The cells were washed with PBS. The AO/EB staining solution was prepared in PBS containing 5 µg/mL of acridine orange and 3 µg/mL of ethidium bromide. In total, 100 µL of staining solution was added to each well and incubated for 5 min at room temperature in the dark. After the incubation, the cells were observed using a ZOE^TM^ fluorescent cell imager. The live (green) and dead (red) cells were counted using ImageJ software. The viability percentage was calculated using the formula

$$Cell\;viability\;(\%)=\frac{Number\;of\;live\;cells}{\left(Number\;of\;live\;cells+Number\;of\;dead\;cells\right)}\times 100$$

### 4.18. Murine Model and In Vivo Study

Age-matched male Balb/C mice (6 weeks old; 15–18 g) were procured from Spring Labs (Tumakuru, Karnataka, India) and housed at the Central Laboratory Animal Facility at Yenepoya (Deemed to be University), Deralakatte, Mangalore, India. All procedures were carried out in accordance with the guidelines approved by the Yenepoya University Institutional Animal Ethics Committee (Approval number: YU/IAEC/P03/2025). Mice were maintained under specific pathogen-free conditions, with a 12 h light/dark cycle, and had free access to water and food throughout the study. A *P. aeruginosa* secretome-associated acute lung inflammation mouse model was established by the intranasal instillation of *P. aeruginosa* secretome. Twenty-two mice were randomly assigned to five experimental groups, with comparable body weights: (1) PBS control (*n* = 4), (2) PAO1 secretome (100 µg/kg; *n* = 5), (3) PAO1 secretome (100 µg/kg) followed immediately by ISL (2.5 mg/kg; *n* = 4), (4) RS85 secretome (100 µg/kg; *n* = 5), (5) RS85 secretome (100 µg/kg) followed immediately by ISL (2.5 mg/kg; *n* = 4). The larger sample sizes in the secretome-only groups were selected to account for potential mortality during the experimental period. The control group received intranasal PBS. All the treatments were administered intranasally once daily for seven consecutive days. On the 8th day, mice were euthanized with ketamine (300 mg/kg) and xylazine (30 mg/kg) via intraperitoneal (i.p.) injection, and peripheral blood (500 µL) and lung tissues were collected for downstream analyses. The group allocation was partially blinded to avoid any bias in the data analysis.

Neutrophil-rich leukocytes were isolated from the mouse peripheral blood following centrifugation and ammonium chloride–potassium (ACK)-mediated red blood cell (RBC) lysis protocol [73]. The peripheral blood was centrifuged at 3000 rpm for 10 min to separate plasma, the buffy coat, and erythrocytes. Leukocytes were isolated from the buffy coat and incubated with ammonium chloride–potassium (ACK) lysis buffer (5 volumes, 5 min, room temperature) to remove residual erythrocytes. Lysis was halted by dilution with PBS (1:10), followed by centrifugation at 1500 rpm for 5 min at 4 °C. The supernatant was discarded, and the pellet was resuspended in PBS. Given the strong neutrophilic inflammatory response induced by bacterial secretome, the leukocyte population obtained was expected to be enriched in neutrophils; however, no additional neutrophil-specific purification was performed.

Extracellular DNA release was assessed in neutrophil-rich leukocyte preparations using Hoechst staining, following previously reported methods with a few modifications [74]. Briefly, 50 µL of isolated leukocytes (1 × 10^5^ cells/well) suspended in PBS were seeded into a 96-well plate and allowed to adhere for 20 min at room temperature. Cells were subsequently fixed with 4% PFA for 15 min and washed with PBS. Nuclear and extracellular DNA was stained with Hoechst dye (5 µM) and observed using a ZOE^TM^ fluorescence imager.

Immunofluorescence staining was performed as per the previously described method with minor modifications [14]. Briefly, isolated leukocytes (1 × 10^5^ cells/well) suspended in PBS were seeded and adhered to a 96-well plate. Subsequently, cells were fixed with 4% PFA for 15 min and washed with PBS. Following fixation, cells were permeabilized and blocked for 1 h at room temperature using 5% bovine serum albumin (BSA) and 0.3% Triton X-100 to prevent non-specific binding. For NET assessment, cells were incubated overnight at 4 °C with goat anti-myeloperoxidase (MPO) antibody (1:100; R&D Systems, AF3667) and rabbit anti-NE antibody (1:100; ABclonal Technology, Woburn, MA, USA; A13015). After primary antibody incubation, cells were washed and incubated with secondary antibodies, such as Alexa Fluor 488 donkey anti-goat or Alexa Fluor 647 donkey anti-rabbit (1:1000, Invitrogen). DNA was counterstained using Hoechst dye (5 µM). Images were acquired using the ZOE^TM^ fluorescent cell imager.

Lung tissue paraffin sections were stained with hematoxylin and eosin (H&E). Histopathological evaluation was performed on the lung sections. Inflammatory severity and tissue damage were independently scored in a blinded manner on a scale from 0 (normal histology) to 5 (severe pathology) for perivascular, peribronchial and parenchymal inflammation, as well as epithelial injury. Scores for individual parameters were aggregated to generate a composite histological score (range: 0–20). Alveolar structural changes were quantified by measuring MLI, which is defined as the ratio of total alveolar septal length to the number of alveoli per microscopic field [14].

Lung tissue sections were deparaffinized and rehydrated prior to antigen retrieval by heating in 0.01 M citrate buffer (pH 6.0) at 95 °C for 10 min. Autofluorescence in paraffin-embedded tissue was minimized by treating the sections with sodium borohydride (1 mg/mL in ice-cold PBS) for three consecutive 10-minute incubations. To quantify NETs, slides were incubated overnight at 4 °C with goat anti-myeloperoxidase (MPO) antibody (1:50; R&D Systems, AF3667) and rabbit anti-histone H3 citrullinated (Cit H3) antibody (1:100; Abcam, Cambridge, UK; ab5103), followed by incubation with appropriate species-specific secondary fluorescent antibodies (Alexa Fluor 488 donkey anti-goat or Alexa Fluor 647 donkey anti-rabbit, 1:500, Invitrogen). DNA was stained with 50 µM Hoechst dye and mounted with 90% glycerol.

### 4.19. Molecular Docking of ISL with Bacteria QS System and NADH Oxidase of Neutrophils

The receptor proteins used for the molecular interaction study were identified based on earlier studies [40]. Molecular docking analysis was used to evaluate the binding interactions between ISL and the selected target proteins. Molecular docking analysis was conducted using PyRx version 0.9.8 [75]. The three-dimensional (3D) structures of ISL and the target proteins of bacterial QS system and the ROS-dependent NETosis pathway were obtained from the PubChem (https://pubchem.ncbi.nlm.nih.gov/) and Protein Data Bank (PDB) (https://www.rcsb.org/ accessed on 6 July 2026) databases, respectively. The corresponding PDB ID was shown in Table 2. Before docking, the active binding site of the receptor proteins was defined by FastDRH [76]. The docking grid was defined based on the binding site of each receptor. Molecular docking was performed using PyRx’s default docking parameters to generate the best binding pose. The docking complexes were observed using Discovery Studio 2020 Client and UCSF Chimera version 1.10.1 [77]. All the docked conformations were sorted by binding affinity between ISL and the target receptors.

### 4.20. Statistics and Reproducibility

Statistical analyses were performed using GraphPad Prism (Version 8). Data are presented as mean ± standard deviation. Each in vitro experiment included five independent biological replicates (donors) with two technical replicates per donor. For microscopy-based analyses, five regions of interest (ROIs) were analyzed per sample. Technical replicates and ROI measurements were averaged to obtain a single value for each biological replicate, and only these biological replicate values were used as independent observations for statistical analyses. Comparisons among multiple groups were performed using one-way analysis of variance (ANOVA), while two-way ANOVA was used for experiments involving two independent variables. Appropriate post hoc tests (Tukey’s multiple comparisons test) were applied wherever necessary.

## 5. Conclusions

This study demonstrates that ISL may modulate both bacterial virulence and host inflammatory responses by attenuating QS-regulated virulence phenotypes in *P. aeruginosa* and modulating neutrophil-mediated inflammatory responses, including ROS generation and NET formation. These findings highlight its potential as a candidate for targeting bacterial virulence and host inflammatory responses in *P. aeruginosa*-associated infections. However, the use of a single clinical *P. aeruginosa* isolate from a patient with COPD and neutrophils from healthy donors warrants further validation in the larger COPD cohort, and also consideration of inter-donor variability. In addition, the predicted molecular interactions require experimental verification to be corroborated and to support further mechanistic inferences and potential applications.

## Figures and Tables

**Table 1:** The zone of inhibition (mm) representing antibacterial and anti-QS activity of ISL against COPD isolate *P. aeruginosa* RS85 and reference strains *C. violaceum* ATCC 12472 and *P. aeruginosa* PAO1. Values are mean ± SD (*n* = 3).

	*C. violaceum* ATCC 12472		*P. aeruginosa* PAO1		*P. aeruginosa* RS85	
	Antibacterial	AQS	Antibacterial	AQS	Antibacterial	AQS
ISL	13 ± 1	8 ± 0.25	–	16 ± 0.5	–	18 ± 0.5
3H-furanone	9 ± 0.5	7 ± 0.5	–	14 ± 0.5	–	12 ± 0.5
Ciprofloxacin	43 ± 0.5	–	34 ± 2.5	–	33 ± 0.5	–
DMSO	–	–	–	–	–	–

**Table 2:** Molecular interaction of isoliquiritigenin (ISL) with the bacterial QS system (LasR and RhlR) and the NADPH oxidase/NOX2 organizer (p47phox) and NADPH oxidase 2 (NOX2) of neutrophils.

Proteins	PDB Code	Ligand	Hydrogen Bond	Binding Affinity (kcal/mol)
LasR	6MWH	ISL	THR75; TYR93; SER129	−11.1
RhlR	2Q0O	ISL	LYS167	−7.4
p47phox	1KQ6	ISL	ARG41; GLU44; GLU47; ASP119	−7.787
NOX2	8GZ3	ISL	PHE145; ARG147; GLU215	−8.062

## Data Availability

The Whole Genome Shotgun project for *Pseudomonas aeruginosa* RS85 has been deposited at DDBJ/ENA/GenBank under the accession number JBXXKE000000000. The raw reads have been deposited at GenBank under the BioProject ID and SRA accession number for strain RS85: PRJNA1457228 and BioSample accession: SAMN57450392. The authors declare that all information required to support the study’s conclusions is included in this paper and in the Supplementary Materials.

## Supplementary Materials

The following supporting information can be downloaded at: https://www.mdpi.com/article/10.3390/molecules31214007/s1, Table S1. Identification of N-acyl homoserine lactones (AHLs) in P. aeruginosa strains PAO1 and RS085 by LC–MS analysis. Table S2: Comparative analysis of IC_50_ values obtained for isoliquiritigenin (ISL) to ascorbic acid (standard) in ABTS and DPPH radical scavenging assays. Figure S1: Synthetic acyl-homoserine lactone induces NET formation in human neutrophils (a) Fluorescence images of neutrophils stained with Hoechst 33342, showing the release of extracellular DNA after stimulation with AHLs, N-butanoyl-L-homoserine lactone (C4-HSL), N-dodecanoyl-L-homoserine lactone (C12-HSL), or N-(3-oxododecanoyl)-L-homoserine lactone (3-OC12-HSL). (b) Quantification of fluorescence intensity corresponding to extracellular DNA following AHL stimuli. Figure S2. Magnified views depicting the influence of cells and secretome of tested P. aeruginosa strains on NETosis and the antioxidant response of ISL. Zoomed-in sections from Figure 2, depicting a close-up view of neutrophils challenged with the cells of P. aeruginosa RS85, PAO1 and PAO1-JP2 and corresponding secretome (RS85 sup, PAO1 sup and PAO1-JP2 sup) on NETosis (red triangles). Upper row: untreated neutrophils (a) and neutrophils treated with cells of PAO-JP2 (b), PAO1 (c) and RS85 (d); Lower row: neutrophils treated with PMA (e), and secretomes of PAO-JP2 (f), PAO1 (g) and RS85 (h); Scale bar, 40 μm. Antioxidant potential of ISL by ABTS radical decolourization assay (i) and DPPH radical scavenging activity (j). Concentration-dependent curves of radical scavenging activity with ISL are shown. Ascorbic acid served as a positive standard. Figure S3: Differential intracellular ROS generation in neutrophils activated by cells of P. aeruginosa strains (RS85 and PAO1) and corresponding secretome. (a) Representative images by fluorescence microscopy of intracellular DCFH-DA-stained neutrophils in the presence of P. aeruginosa PAO1 and RS85 cells (BC) or their corresponding cell-free supernatants (sup). The negative control was non-stimulated cells, while the positive control was PMA-stimulated cells (for ROS production). Green colored represents the ROS produced within the cells after oxidation of DCFH-DA. Merged images showed that the fluorescence image overlapped the bright-field image. (b) ROS production within neutrophils is expressed as the percentage of fluorescence intensity relative to the untreated control. Figure S4. ISL-driven suppression of intracellular ROS generation in neutrophils activated by cells and supernatants of P. aeruginosa RS85 and PAO1. Representative images by fluorescence microscopy of intracellular DCFH-DA-stained neutrophils stimulated with the respective cells along with ISL (a). Quantitative analysis of intracellular ROS production during respective treatments (b). Representative images by fluorescence microscopy of intracellular DCFH-DA-stained neutrophils stimulated with the respective secretome along with ISL (c). Quantitative analysis of intracellular ROS production during the respective treatments (d). Figure S5. Immunofluorescence analysis of NET formation in P. aeruginosa stimulated neutrophils following ISL intervention. Representative fluorescence images of neutrophils (a,b) stained with Hoechst (blue), myeloperoxidase (MPO; green) and neutrophil elastase (NE; red) and merged images. Neutrophils pretreated with NAC prior to PAO1 stimulation and treated with ISL alone served as controls. ISL intervention resulted in reduced extracellular DNA release and co-localization of MPO and NE with DNA, indicating NET formation. The accompanying bar graphs (c,d) show a quantitative analysis of NET-associated fluorescence intensity. Figure S6. Neutrophil viability assay following ISL treatment using MTT and acridine orange/ethidium bromide (AO/EB) staining. (a) MTT assay showing neutrophil viability following exposure to different concentrations of ISL. (b) Fluorescent images of AO/EB-stained neutrophils, showing viable cells with green fluorescence and non-viable cells with orange/ red fluorescence. (c) Bar graph showing viability percentage based on AO/EB staining.
