# Supplementary material for: Isoliquiritigenin Modulates Quorum Sensing-Associated Neutrophil Extracellular Trap Formation in COPD Isolate Pseudomonas aeruginosa
Source: Molecules. 2026 Nov 6;31(21):4007. doi: 10.3390/molecules31214007 (PMC13764961; doi:10.3390/molecules31214007)
Supplement: Supplementary file 1 [file molecules-31-04007-s001.zip › molecules-4625283-Supplementary file_ISL.pdf]

Supplementary file

Isoliquiritigenin modulates quorum sensing-associated neutrophil extracellular trap formation in COPD isolate *Pseudomonas aeruginosa*

Honagodu Ravichandra Dhanyashree[1], Rakshitha Charavu[2], Asif Hameed[1,3,4]*, Kizhakkumkara Karthika[1], Adarsh B. Mynalli[5], Suprith Surya[6], Yashodhar Prabhakar Bhandary[2] and Rajesh P. Shastry[1,3,4]*

[1]*Division of Microbiology and Biotechnology, Yenepoya Research Centre, Yenepoya (Deemed to be University), Deralakatte, Mangalore 575018, India*

[2]*Division of Cell Biology and Molecular Genetics, Yenepoya Research Centre, Yenepoya (Deemed to be University), Deralakatte, Mangalore 575018, India*

[3]*Anaerobic & Translational Genomics Centre (ATGC), Yenepoya Research Centre, Yenepoya (Deemed to be University), Deralakatte, Mangalore-575018, India*

[4]*Caenorhabditis elegans Laboratory Facility (CeLF), Yenepoya Research Centre, Yenepoya (Deemed to be University), Deralakatte, Mangalore-575018, India*

[5]*Department of Respiratory Medicine, Yenepoya Medical College Hospital, Deralakatte, Mangalore-575018, India*

[6]*Advanced Surgical Skill ENhancement Division (ASSEND), Yenepoya (Deemed to be University), Deralakatte, Mangalore-575018, India*

Table S1. Identification of N-acyl homoserine lactones (AHLs) in *P. aeruginosa* strains PAO1 and RS085 by LC–MS analysis

**Strains**	**Identity**	**Observed *m/z***
**PAO1**	3-Oxo-12 HSL	298.64
	C4 HSL	171.19
**RS085**	3-Oxo-C12 HSL	298.41
	3-OH-C10 HSL	273.38
	C4 HSL	172.18

Table S2: Comparative analysis of IC_{50} values obtained for isoliquiritigenin (ISL) compared with ascorbic acid (standard) in ABTS and DPPH radical scavenging assays.

**Sample**	**IC_{50} (µg/mL)**	
	**ABTS**	**DPPH**
Ascorbic acid	14.56	0.4486
Isoliquiritigenin	0.8970	51.19

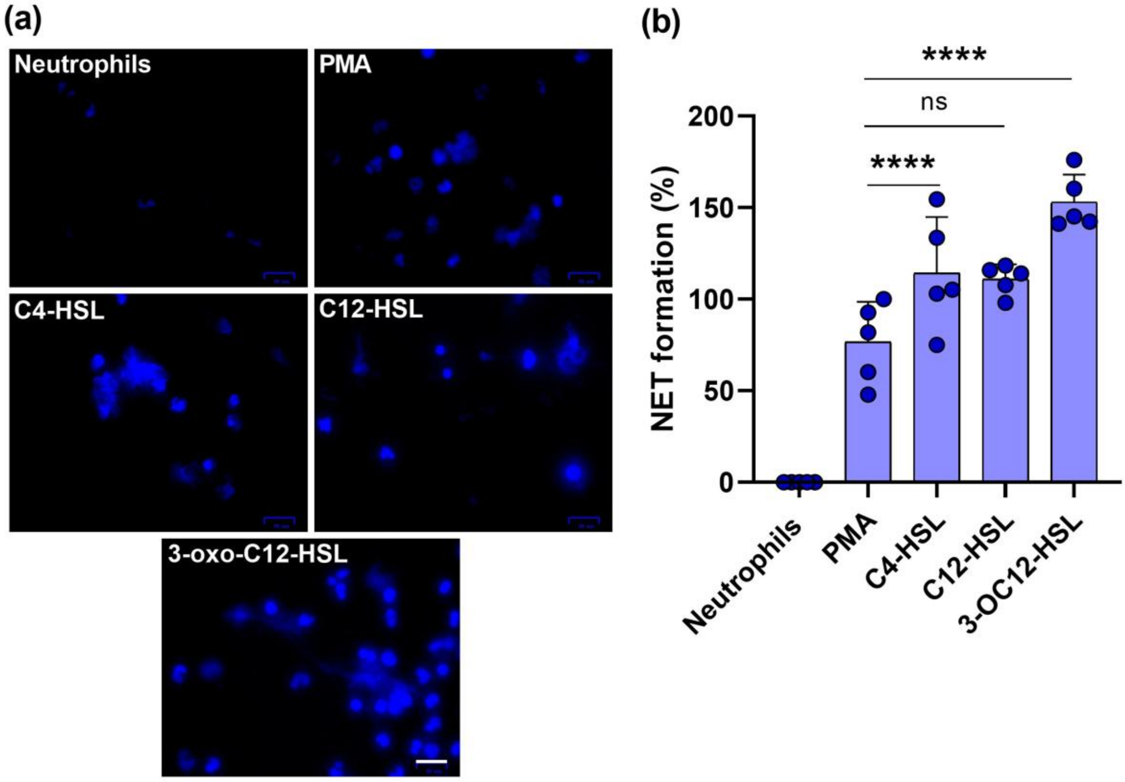

Figure S1: Synthetic acyl-homoserine lactone induces NET formation in human neutrophils (a) Fluorescence images of neutrophils stained with Hoechst 33342, showing the release of extracellular DNA after stimulation with AHLs, N-butanoyl-L-homoserine lactone (C4-HSL), N-dodecanoyl-L-homoserine lactone (C12-HSL), or N-(3-oxododecanoyl)-L-homoserine lactone (3-OC12-HSL). (b) Quantification of fluorescence intensity corresponding to extracellular DNA following AHL stimuli. Data are presented as mean ± SD from three biological replicates, with two technical replicates for each experimental condition. Five regions of interest (ROIs) were analysed per sample. Statistical significance was determined using one-way ANOVA followed by Tukey's multiple comparisons test. Level of significance: ns, not significant; ****$p<0.0001$. Scale bar, 25 μm.

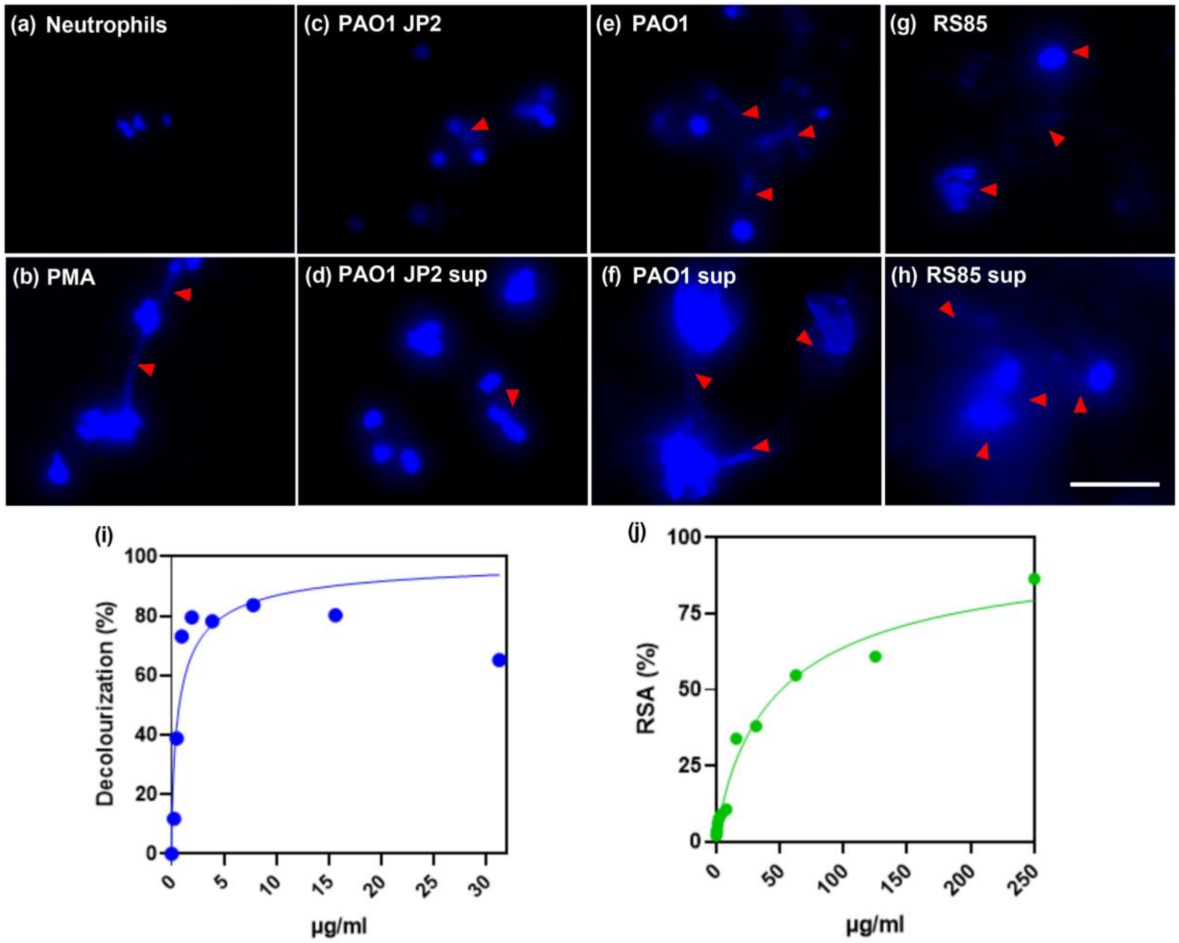

Figure S2. Magnified views depicting the influence of cells and secretome of tested *P. aeruginosa* strains on NETosis and the antioxidant response of ISL. Zoomed-in sections from Figure 2, depicting a close-up view of neutrophils challenged with the cells of *P. aeruginosa* RS85, PAO1 and PAO1-JP2 and corresponding secretome (RS85 sup, PAO1 sup and PAO1-JP2 sup) on NETosis (red triangles). Upper row: untreated neutrophils (a) and neutrophils treated with cells of PAO-JP2 (b), PAO1 (c) and RS85 (d); Lower row: neutrophils treated with PMA (e), and secretomes of PAO-JP2 (f), PAO1 (g) and RS85 (h); Scale bar, 40 µm. Antioxidant potential of ISL by ABTS radical decolourization assay (i) and DPPH radical scavenging activity (j). Concentration-dependent curves of radical scavenging activity with ISL are shown. Ascorbic acid served as a positive standard.

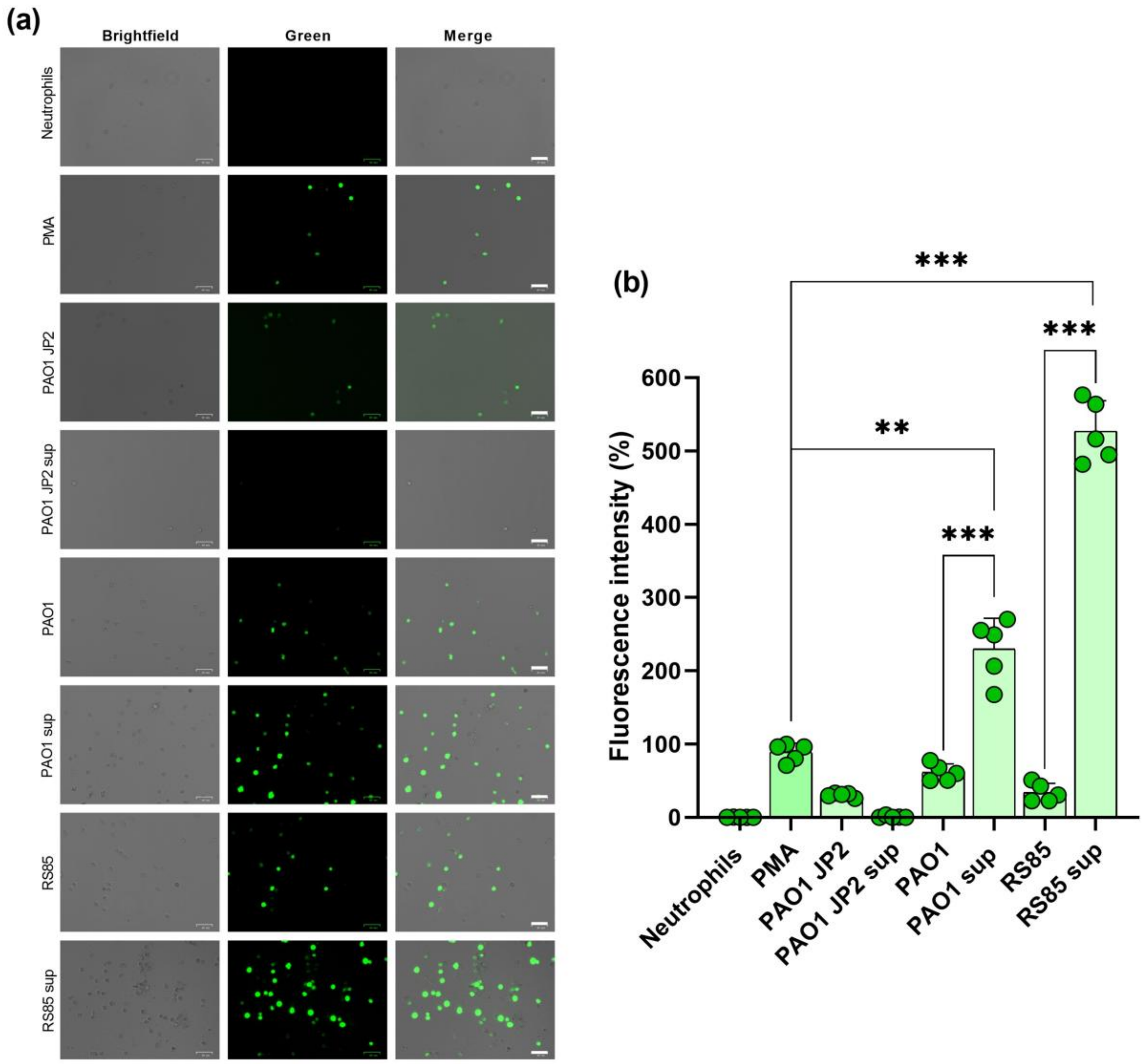

Figure S3: Differential intracellular ROS generation in neutrophils activated by cells of *P. aeruginosa* strains (RS85 and PAO1) and corresponding secretome. (a) Representative images by fluorescence microscopy of intracellular DCFH-DA-stained neutrophils in the presence of *P. aeruginosa* PAO1 and RS85 cells (BC) or their corresponding cell-free supernatants (sup). The negative control was non-stimulated cells, while the positive control was PMA-stimulated cells (for ROS production). Green colored represents the ROS produced within the cells after oxidation of DCFH-DA. Merged images showed that the fluorescence image overlapped the bright-field image. (b) ROS production within neutrophils is expressed as the percentage of fluorescence intensity relative to the untreated control. Data are presented as mean ± SD from three biological replicates, with two technical replicates for each experimental condition. Five regions of interest (ROIs) were analysed per sample. Level of significance: **p<0.01, ***p<0.001. Scale bar, 40 μm.

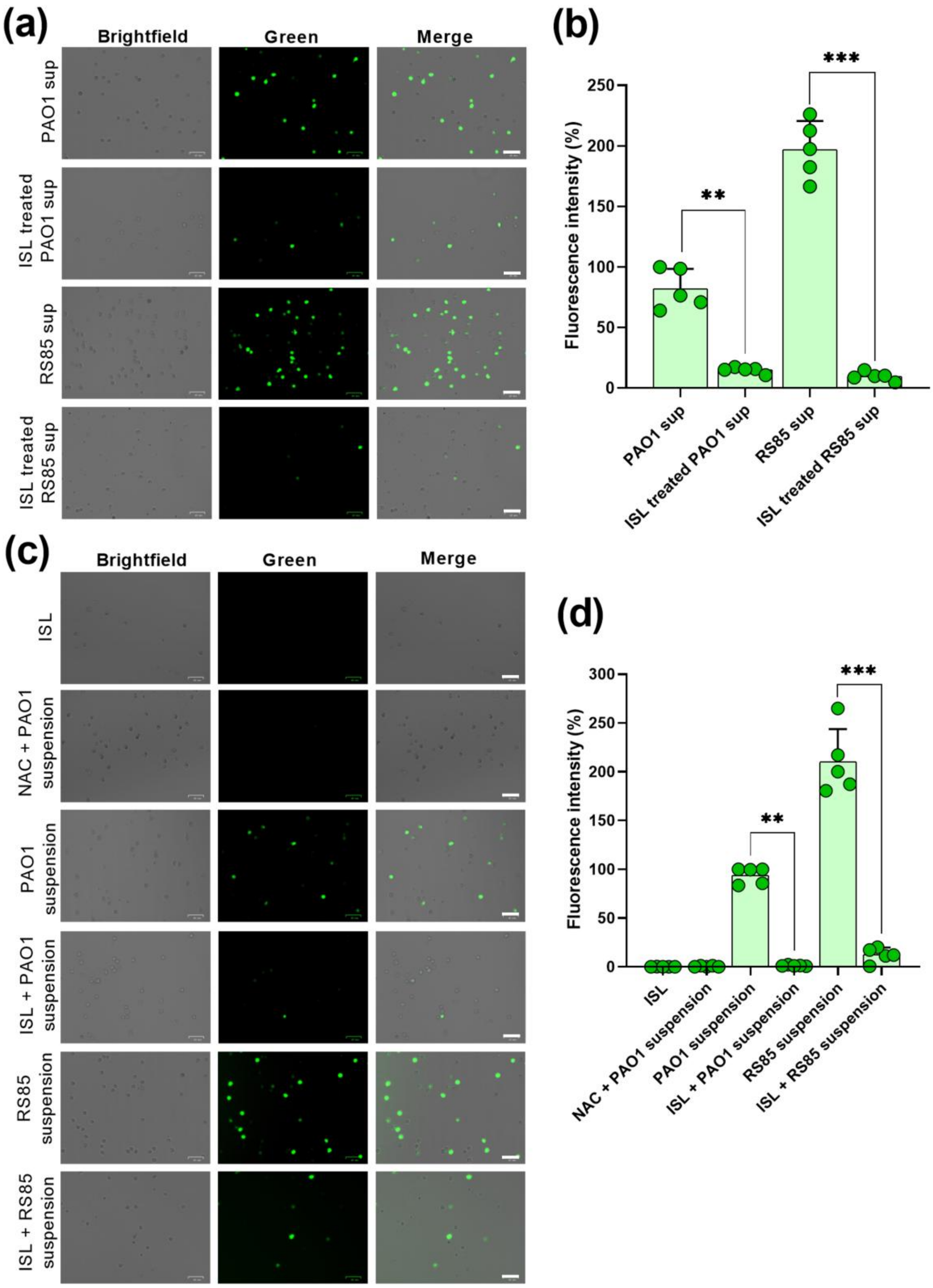

Figure S4. ISL-driven suppression of intracellular ROS generation in neutrophils activated by cells and supernatants of *P. aeruginosa* RS85 and PAO1. Representative images by fluorescence microscopy of intracellular DCFH-DA-stained neutrophils stimulated with the respective cells along with ISL (a). Quantitative analysis of intracellular ROS production during

respective treatments (b). Representative images by fluorescence microscopy of intracellular DCFH-DA-stained neutrophils stimulated with the respective secretome along with ISL (c). Quantitative analysis of intracellular ROS production during the respective treatments (d). Data are presented as mean ± SD from three biological replicates, with two technical replicates for each experimental condition. Five regions of interest (ROIs) were analysed per sample. Level of significance: **p<0.01, ***p<0.001. Scale bar, 40 μm.

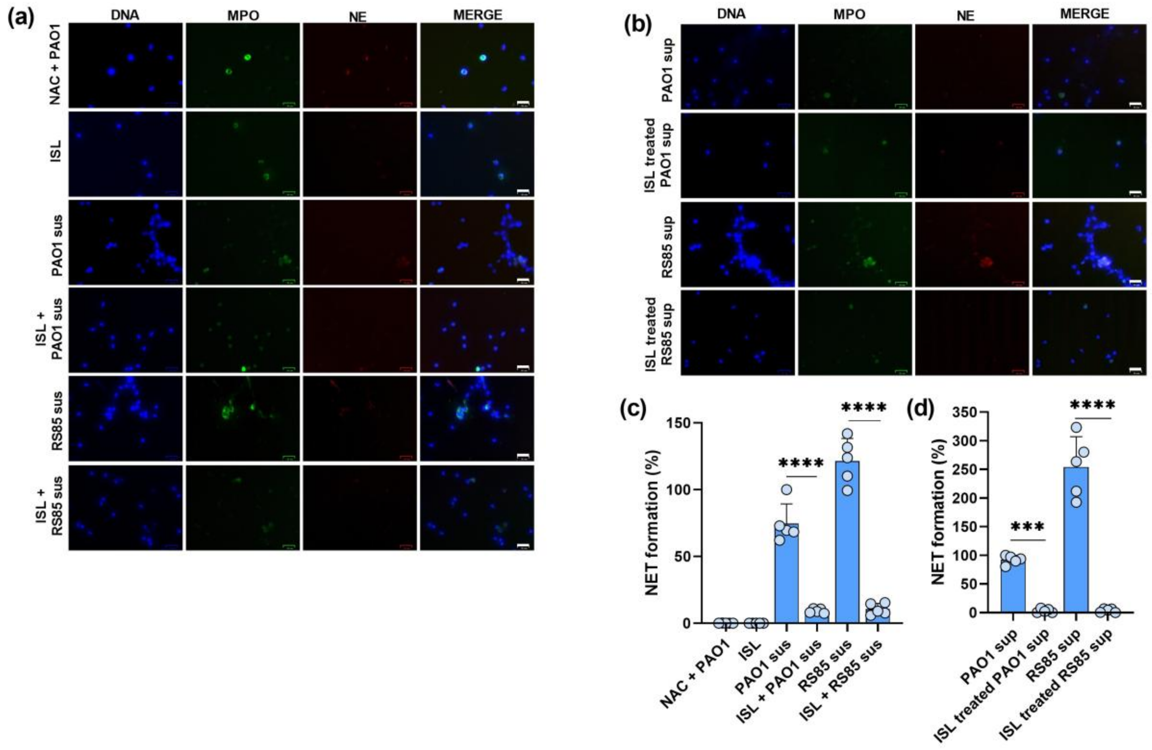

Figure S5. Immunofluorescence analysis of NET formation in *P. aeruginosa* stimulated neutrophils following ISL intervention. Representative fluorescence images of neutrophils (a,b) stained with Hoechst (blue), myeloperoxidase (MPO; green) and neutrophil elastase (NE; red) and merged images. Neutrophils pretreated with NAC prior to PAO1 stimulation and treated with ISL alone served as controls. ISL intervention resulted in reduced extracellular DNA release and co-localization of MPO and NE with DNA, indicating NET formation. The accompanying bar graphs (c,d) show a quantitative analysis of NET-associated fluorescence intensity. Data are presented as mean ± SD. Five regions of interest (ROIs) were analysed per sample, with five biological replicates and two technical replicates for each experimental condition. Statistical significance was determined using one-way ANOVA followed by Tukey's multiple comparisons test. Level of significance: ***p<0.001, ****p<0.0001. Scale bar, 25 μm.

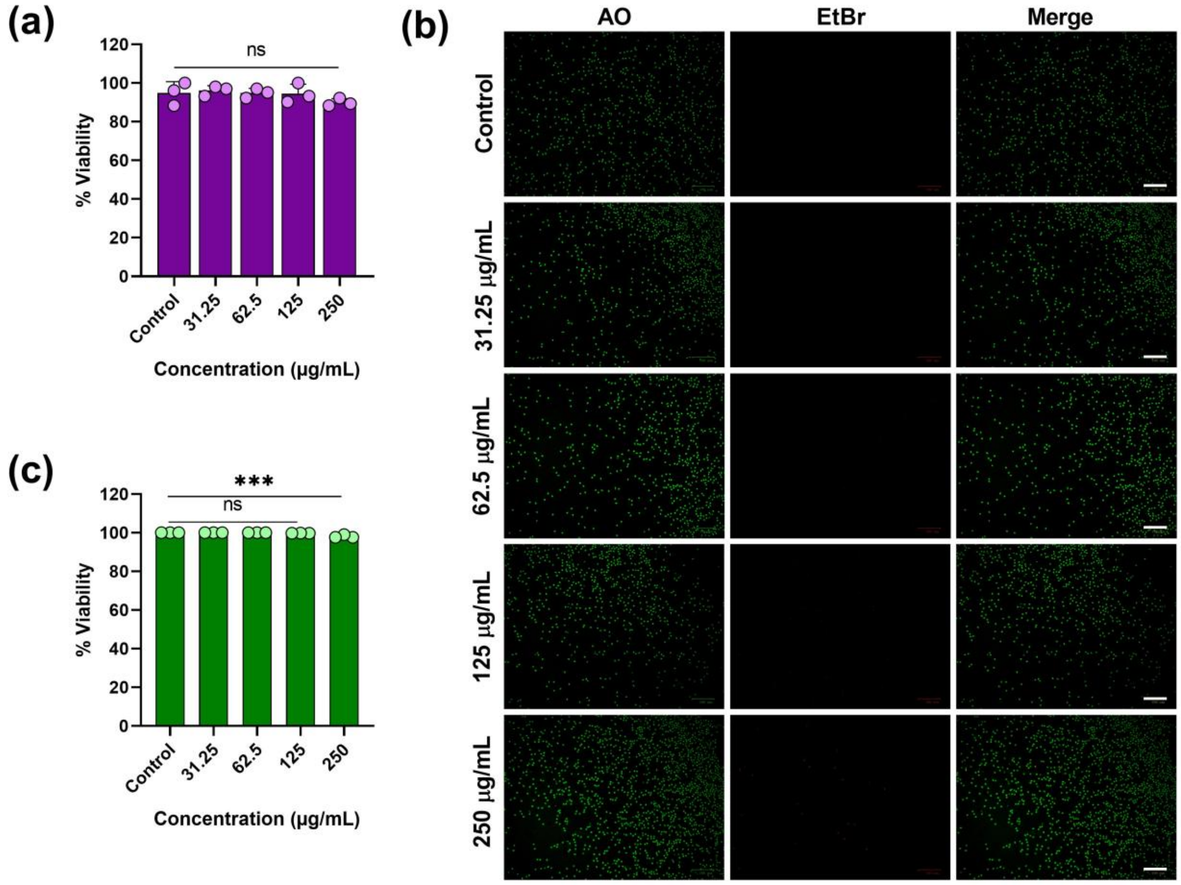

Figure S6. Neutrophil viability assay following ISL treatment using MTT and acridine orange/ethidium bromide (AO/EB) staining. (a) MTT assay showing neutrophil viability following exposure to different concentrations of ISL. (b) Fluorescent images of AO/EB-stained neutrophils, showing viable cells with green fluorescence and non-viable cells with orange/ red fluorescence. (c) Bar graph showing viability percentage based on AO/EB staining. Data are presented as mean ± SD. Three biological replicates and two technical replicates for each experimental condition. Statistical significance was determined using one-way ANOVA followed by Tukey's multiple comparisons test. Level of significance: ns, not significant; ***$p<0.001$. Scale bar, 100 μm.
